# Mutations in *MAB21L2* Result in Ocular Coloboma, Microcornea and Cataracts

**DOI:** 10.1371/journal.pgen.1005002

**Published:** 2015-02-26

**Authors:** Brett Deml, Ariana Kariminejad, Razieh H. R. Borujerdi, Sanaa Muheisen, Linda M. Reis, Elena V. Semina

**Affiliations:** 1 Department of Pediatrics and Children’s Research Institute at the Medical College of Wisconsin and Children’s Hospital of Wisconsin, Milwaukee, Wisconsin, United States of America; 2 Department of Cell Biology, Neurobiology and Anatomy, Medical College of Wisconsin, Milwaukee, Wisconsin, United States of America; 3 Kariminejad-Najmabadi Pathology and Genetics Center, Tehran, Iran; 4 Qom Welfare Organization, Qom, Iran; Stanford University School of Medicine, UNITED STATES

## Abstract

Ocular coloboma results from abnormal embryonic development and is often associated with additional ocular and systemic features. Coloboma is a highly heterogeneous disorder with many cases remaining unexplained. Whole exome sequencing from two cousins affected with dominant coloboma with microcornea, cataracts, and skeletal dysplasia identified a novel heterozygous allele in *MAB21L2*, c.151 C>G, p.(Arg51Gly); the mutation was present in all five family members with the disease and appeared de novo in the first affected generation of the three-generational pedigree. *MAB21L2* encodes a protein similar to *C. elegans* mab-21 cell fate-determining factor; the molecular function of MAB21L2 is largely unknown. To further evaluate the role of *MAB21L2*, zebrafish mutants carrying a p.(Gln48Serfs*5) frameshift truncation (mab21l2^Q48Sfs*5^) and a p.(Arg51_Phe52del) in-frame deletion (*mab21l2^R51_F52del^*) were developed with TALEN technology. Homozygous zebrafish embryos from both lines developed variable lens and coloboma phenotypes: mab21l2^Q48Sfs*5^ embryos demonstrated severe lens and retinal defects with complete lethality while *mab21l2^R51_F52del^* mutants displayed a milder lens phenotype and severe coloboma with a small number of fish surviving to adulthood. Protein studies showed decreased stability for the human p.(Arg51Gly) and zebrafish p.(Arg51_Phe52del) mutant proteins and predicted a complete loss-of-function for the zebrafish p.(Gln48Serfs*5) frameshift truncation. Additionally, in contrast to wild-type human *MAB21L2* transcript, mutant p.(Arg51Gly) mRNA failed to efficiently rescue the ocular phenotype when injected into mab21l2^Q48Sfs*5^ embryos, suggesting this allele is functionally deficient. Histology, immunohistochemistry, and in situ hybridization experiments identified retinal invagination defects, an increase in cell death, abnormal proliferation patterns, and altered expression of several ocular markers in the *mab21l2* mutants. These findings support the identification of *MAB21L2* as a novel factor involved in human coloboma and highlight the power of genome editing manipulation in model organisms for analysis of the effects of whole exome variation in humans.

## Author Summary

Coloboma is a hole or gap in one or more of the structures of the eye. Coloboma occurs when the eye is not formed properly during prenatal development. It is often associated with additional eye abnormalities and can result in significant loss of vision. Identification of the genetic causes of coloboma provides more information about how the eye develops. We used whole exome sequencing in an affected family to identify mutations in a new gene associated with dominant coloboma in humans, *MAB21L2*. We used genome editing to disrupt the *mab21l2* gene in zebrafish, which led to similar eye conditions in affected fish, providing additional evidence for the role of this gene in eye development. The function of MAB21L2 is largely unknown; further study of the pathways affected by MAB21L2 deficiency and study of the zebrafish generated by this project will provide more information about the role of this gene in eye development.

## Introduction

Coloboma is a congenital segmental ocular defect which can affect one or more structures of the eye; typical coloboma results in an inferior deficiency of iris, chorioretinal, and/or optic nerve tissue [1–3]. Ocular coloboma is believed to result from failure of normal closure of the optic fissure during embryonic eye development, termed optic fissure closure defect (OFCD) [1,2]. Coloboma can occur as an isolated anomaly (simple coloboma) but in most cases it is associated with additional ocular defects, including microphthalmia, cataract, retinal detachment, and ocular motility disorders [1–4]. Microcornea, a reduction in the diameter of the cornea, is common in colobomatous eyes and can be associated with normal or even increased (macrophthalmic) axial length [2,3,5]. Additional systemic anomalies are present in a large proportion of patients with OFCDs including brain, skeletal, cardiac, or urogenital anomalies [3,4].

A number of genes have been associated with coloboma including transcriptional regulators *SOX2*, *OTX2*, *PAX2*, *PAX6*, *CHD7* and *SALL2;* secreted signaling factor-encoding gene *SHH*; members of the transforming growth factor-beta (TGF-beta) superfamily *GDF6* and *GDF3;* members of the retinoic acid synthesis pathway *STRA6* and *ALDH1A3;* as well as membrane porphyrin transporter *ABCB6*, and a member of the HIPPO growth control pathway, *YAP1* [6–19]. Due to the number of genes involved and the large proportion of unexplained cases, whole exome sequencing has been used with increasing frequency to screen families with coloboma and other ocular conditions [16–21]. Since whole exome sequencing generates a large number of variants and often involves genes with unexplored/unknown function, studies in animal models can be invaluable in providing further insight into the functional roles of candidate genes/variants and their possible involvement in the studied phenotype [17,22]. Recent advances in genomic editing technologies using engineered nucleases empowered researchers with tools for the functional exploration of these genes/variants of interest [23].

In this manuscript we present evidence for the role of *MAB21L2* in human coloboma and further investigate its role in vertebrate ocular development by generation and analysis of zebrafish *mab21l2* mutants.

## Results

### Identification of a *MAB21L2* mutation in a human pedigree affected with ocular disease

**Fig 1:**
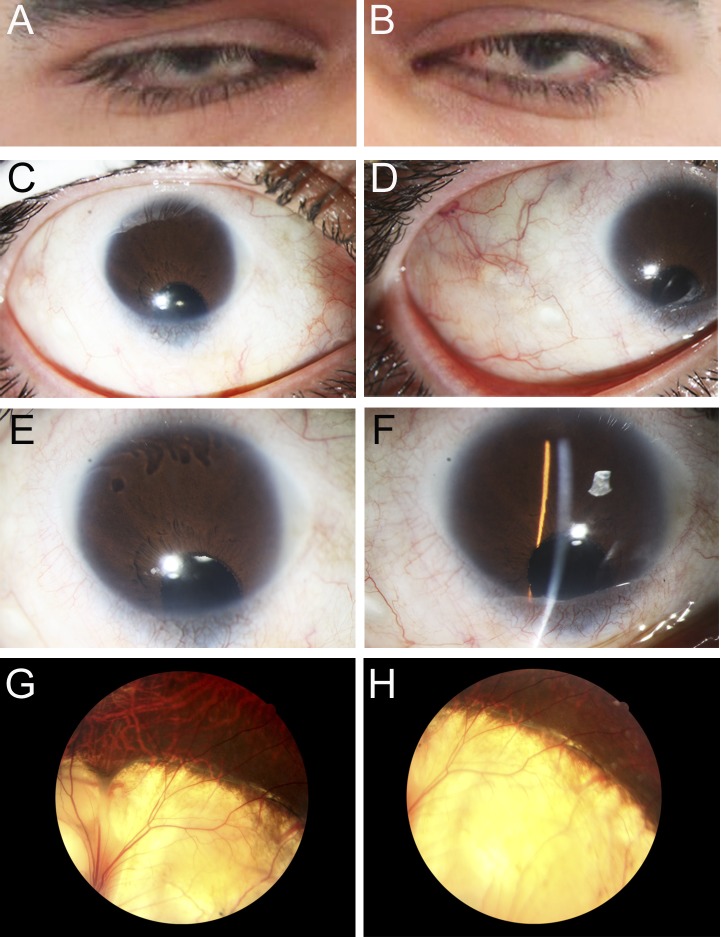
Ocular Images of Patient 1. Photographs of the right (A, C, E, G) and the left (B, D, F, H) eyes showing bilateral iris coloboma with microcornea and corectopia (C-F) as well as chorioretinal coloboma (G, H) in the proband (Patient 1).

**Fig 2:**
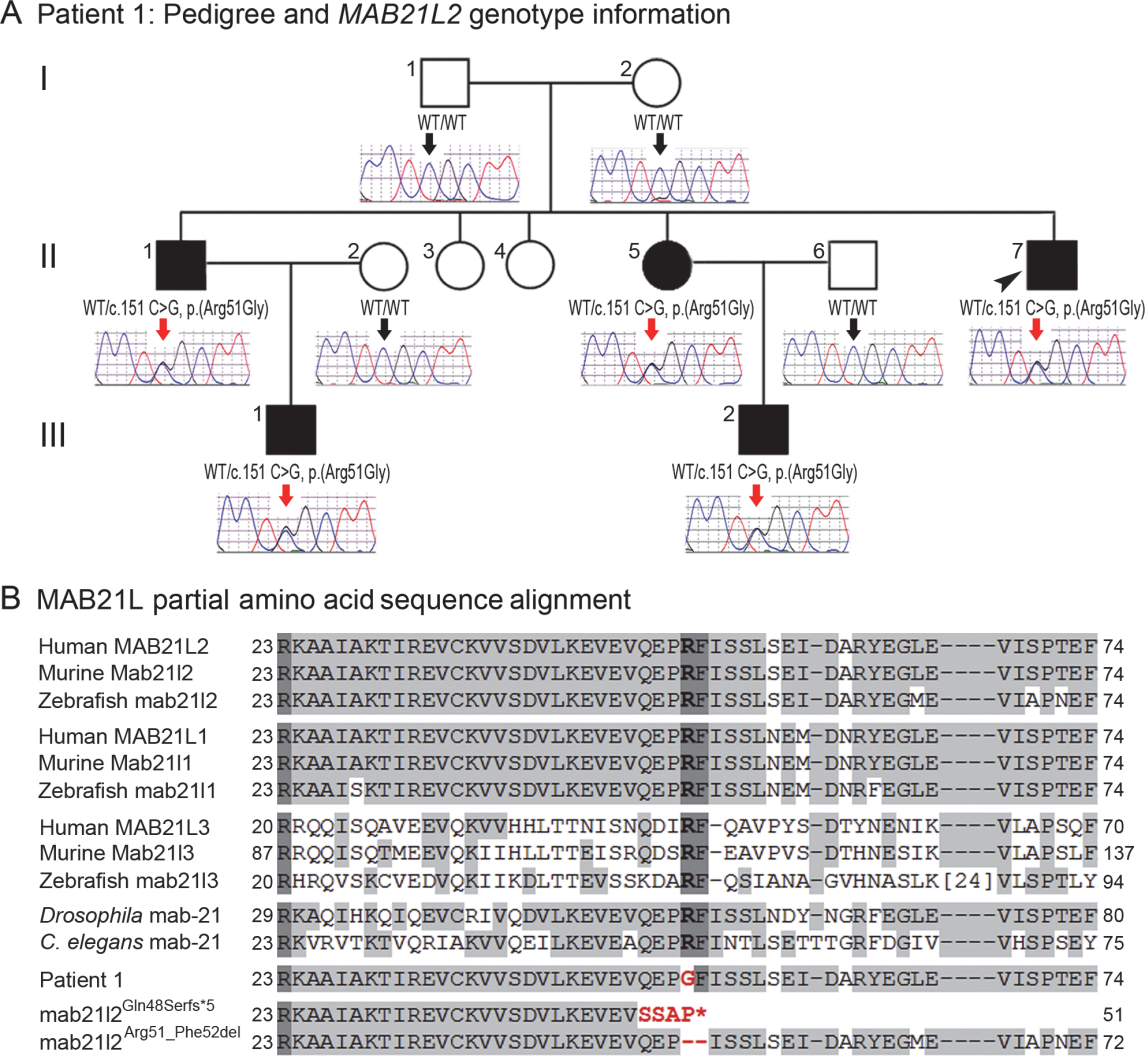
*MAB21L2* mutations and protein sequence conservation. **A.** Three-generation pedigree of Patient 1 with *MAB21L2* genotype information. DNA chromatograms for all tested family members are shown with c.151C position indicated with black (WT allele) or red (heterozygous mutant allele) arrows. The proband (Patient 1) is indicated with a black arrowhead. Please note the presence of the mutant allele in all affected individuals, its absence in unaffected family members, and the presence of a low ‘G’ peak in addition to the normal ‘C’ nucleotide at the mutant position in the proband’s unaffected mother. **B.** Amino acid alignment of the MAB21L1-3 and mab-21 regions surrounding the arginine at position 51; amino acids identical between different homologs are highlighted with a light grey color, three invariant residues are shown in dark grey; the glycine (G) predicted to replace arginine 51 in Patient 1 is shown in red font; the positions and predicted effects of the zebrafish *mab21l2* mutations involving the same region are also shown in red font. Accession numbers for sequences utilized in the alignment are provided in Methods.

The proband (Patient 1) was diagnosed with bilateral microcornea, iris and chorioretinal coloboma, corectopia, nystagmus and cataract (Fig. 1); the cataractous lens of his right eye was removed at age 24. Based on ultrasound measurements at age 32, the axial length was 29.02 mm (right eye) and 24.67 mm (left eye), thus showing no signs of microphthalmia (normal axial length for adult males is approximately 23 mm) [24]. Evaluation of other systems identified mild contracture in the knees and elbows and rhizomelia of the upper and lower limbs: the lengths of the femur (40.3 cm, left and 40.6 cm, right), tibia (34.5cm, left and 34.3 cm, right), humerus (26 cm, left and 26.3 cm, right), ulna (23.5 cm, left and 23.4 cm, right) and radius (22.2 cm, left and 22.6 cm, right) were determined by scanography and the observed femur/tibia (1.18) and humerus/radius (1.17) ratios were found to deviate from the normal reference numbers of 1.23 and 1.31, respectively. No signs of cardiac, brain or other abnormalities were identified. Analysis of family history identified a similarly affected sister, brother, and two nephews, while his father, mother, and two additional sisters were unaffected (Fig. 2A).

Independent whole exome sequencing of DNA samples from the proband’s two affected nephews (III-1 and III-2, Fig. 2A) yielded a mean coverage of 63.9X ± 4.0 for the two samples with 96.25% ± 0.49 of bases having >10X coverage. Initial analysis of the whole exome data excluded mutations in 72 known factors involved in anophthalmia, microphthalmia, and/or coloboma [25]. Comparison of whole exome data of the two nephews identified 16 shared heterozygous variants that were novel or rare (<1%): one splicing, one frameshift indel, one inframe indel, and 13 missense alleles (three novel and ten rare) that were found to be damaging by at least 4 functional effect prediction programs (S1 Table). The five novel alleles and one rare frameshift variant were tested for cosegregation in the pedigree; only the heterozygous *MAB21L2* missense mutation c.151 C>G, p.(Arg51Gly), was present in all five affected individuals (II-1, -5, -7 and III-1, -2) and absent in the unaffected parents (a low ‘G’ peak at position c.151 was seen in the mother’s sample (I-2) in addition to the wild-type ‘C’ on sequencing of two independent PCR reactions, suggesting that she is likely to have low-level mosaicism for this mutation) (Fig. 2A). The *MAB21L2* variant was further identified as a strong candidate based on its absence in all control populations (EVS, dbSNP, and 1000 genomes) and having the highest Genomic Evolutionary Rate Profiling (GERP++) score, as well as enrichment of both human *MAB21L2* and mouse *Mab21l2* transcripts in ocular structures based in BioGPS (http://biogps.org). The mutation was predicted to be damaging by SIFT, Polyphen2, Mutation Taster and MutationAssessor, and demonstrated high conservation scores for the affected nucleotide with a GERP++ score of 6.16 and a PhyloP score of 2.94 (of note, the highest possible GERP++ score is 6.17 (most conserved) [26]).

The *MAB21L2* gene, located at 4q31.3, encodes a 359-a.a. protein similar to *C*. *elegans* mab-21 cell fate-determining factor [27], with the mab-21 domain predicted to span amino acids 62–346 (based on Pfam (Protein FAMilies database) [28]. Alignment of MAB21L amino acid sequences from different species revealed that the arginine residue at position 51 is conserved in all MAB21L human, mouse and zebrafish proteins as well as fruit fly and *C*. *elegans* mab-21 (Fig. 2B). The function and domain structure of MAB21L proteins are largely unknown; the most similar protein with a known structure is human MB21D1 (Mab-21 domain containing 1, also known as cyclic GMP-AMP synthase (cGAS)), a protein involved in innate immunity and binding to cytosolic double stranded DNA to activate interferon production [29]. The domain structure of MB21D1 includes a mab-21 domain and two DNA-binding domains, one located N-terminally to the mab-21 domain (with a three-residue overlap) and the other situated within the C-terminal portion of the mab-21 domain [29,30]. If MAB21L2 structure is similar to MB21D1, then the arginine at position 51 of MAB21L2 would be located in proximity to the predicted mab-21 domain and within the N-terminal DNA-binding domain; this is consistent with the fact that arginine represents the most basic amino acid and is often observed at protein-nucleic acid interaction sites.

### Analysis of additional human patients affected with ocular disease

To further explore the role of *MAB21L2* in human coloboma phenotypes, we examined 276 patients with developmental ocular conditions from our collection as well as whole exome data from 125 cases derived from the UK10K_Rare_Coloboma (EGA Study ID: EGAS00001000127) project of the UK10K Consortium study [18,31]. We identified no additional mutations in our population that included 39 patients affected with A/M and coloboma, 16 patients with coloboma and normal eye size, 104 patients with A/M without coloboma, 50 with aniridia, 37 with various anterior segment dysgenesis conditions, 12 with cataract, and 18 with other developmental ocular conditions. Analysis of the UK10K_Rare_Coloboma cohort identified a heterozygous mutation affecting the same arginine residue described above (Fig. 2B), c.152 G>A, p.(Arg51His), in two samples: UK10K_COL5001067 (the c.152 G>A change was present in 10 out of 23 high quality reads) and UK10K_COL5001068 (the c.152 G>A change was seen in 21 out of 32 high quality reads). While the publicly available UK10K data does not specify family relationships, the presence of identical mutations in consecutively numbered samples suggests that these individuals may be family members. Similar to the c.151 C>G, p.(Arg51Gly) allele, this mutation was not reported in the Exome Variant Server (0/13,006 alleles), the 1000 Genomes Browser (0/2,194 alleles), or dbSNP, and is predicted to be damaging by SIFT, Polyphen2, Mutation Taster and Mutation Assessor. These data indicate a small contribution of *MAB21L2* mutations to human coloboma phenotypes (<2%) and suggest no role for *MAB21L2* mutations in the other ocular disorders that were examined in this study.

### Zebrafish *mab21l2* expression studies

**Fig 3:**
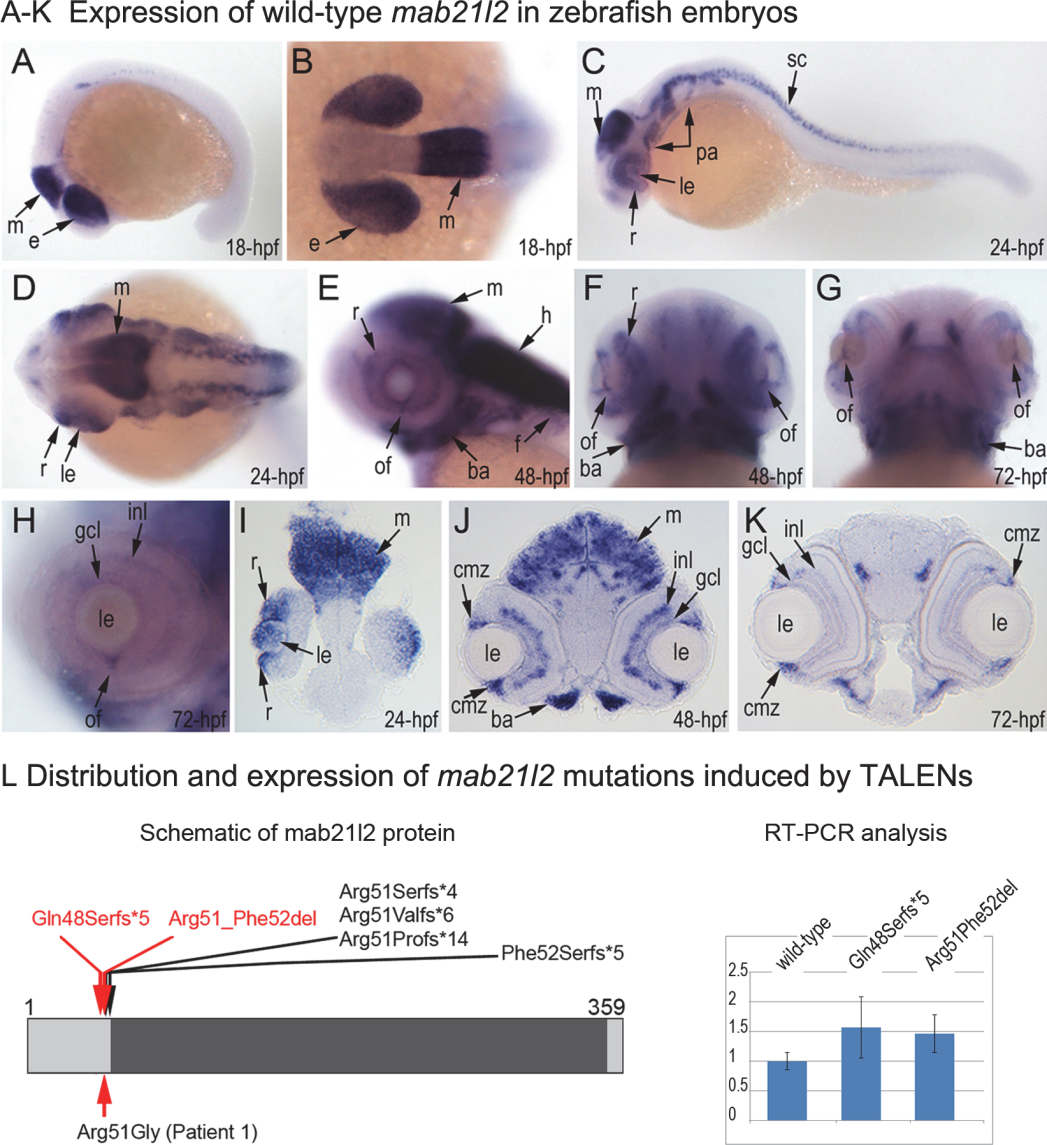
Expression and mutations of zebrafish *mab21l2*. **A-K.** Expression pattern of *mab21l2* in zebrafish 18–72-hpf embryos. Whole mount images (A-H) and sections (I-K) are shown. **A, B.** At 18-hpf, expression in the presumptive eye field (e) and midbrain (m) is observed. **C, D, I.** At 24-hpf, *mab21l2* expression is seen in the periphery of the retina (r), lens (le), spinal cord (sc), midbrain (m) and pharyngeal arch region (pa). **E-H, J, K.** At 48–72-hpf, expression in the ciliary marginal zone (cmz), inner nuclear layer (inl) and ganglion cell layer (gcl) of the retina, and the region of the optic fissure (of) in the eye as well as the midbrain (m), hindbrain (h), developing fins (f), and branchial arches (ba) is shown with arrows. **L.** Distribution and expression of *mab21l2* mutations induced by TALENs. On the left, a schematic of the zebrafish mab21l2 protein is shown as a light grey box with the mab-21 domain (amino acids 62–346) indicated in dark grey color; the positions of the zebrafish mutations identified in the progeny of TALEN-injected fish are shown at the top of the box and the position of the human mutation identified in Patient 1 is indicated at the bottom; the positions of the p.(Gln48Serfs*5) and p.(Arg51_Phe52del) mutations are shown with red arrows. On the right, a graph summarizing results of semi-quantitative RT-PCR analysis of wild-type and mutant *mab21l2* transcript levels in 48-hpf homozygous embryos is shown.

To investigate the role of *MAB21L2/mab21l2* in ocular development, we performed careful evaluation of *mab21l2* expression in zebrafish using in situ hybridization. At 18-hpf, expression was seen in the presumptive eye field and midbrain (Fig. 3A, B). At 24-hpf, *mab21l2* expression was seen in the retina (ventral and dorsal periphery), lens, spinal cord, midbrain and pharyngeal arch region (Fig. 3C, D, I). In 48–72-hpf, the transcripts could be detected in the ciliary marginal zone (CMZ) (or germinal zone) of the retina that contains multipotent retinal progenitors, in the inner nuclear and ganglion cell layers of the retina, and around the optic fissure in the eye, as well as the midbrain, hindbrain, developing fins, and branchial arches (Fig. 3E-H, J, K).

### Generation and gross morphological analysis of *mab21l2* zebrafish mutants

To determine the effect of *mab21l2* deficiency on ocular development, the *mab21l2* gene was disrupted using TALEN genome editing technology. DNA sequencing of *mab21l2* mutant fish identified the following alleles: c.141_153delCCAAGAGCCCCGT, p.(Gln48Serfs*5); c.150_156delCCGTTTC, p.(Arg51Serfs*4); c.151delC, p.(Arg51Valfs*6); c.151dupC, p.(Arg51Profs*14); and c.155delT, p.(Phe52Serfs*5) frameshift mutations (all predicted to result in truncation of the mab21l2 protein at ~14% of its total length) and an in-frame deletion of two amino acids including the arginine at position 51, c.151_156delCGTTTC, p.(Arg51_Phe52del) (Fig. 3L, S1 Fig.).

**Fig 4:**
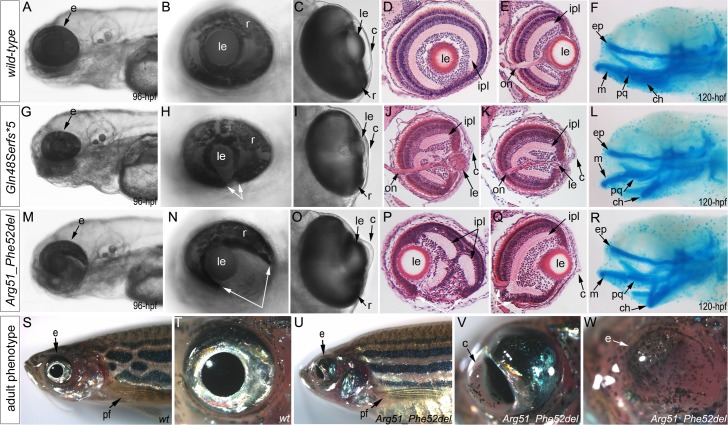
Phenotypic analysis of homozygous embryos carrying mutant *mab21l2* alleles encoding p.(Gln48Serfs*5) truncation (*mab21l2* ^Q48Sfs*5^) and p.(Arg51_Phe52del) in-frame deletion (*mab21l2* ^*R51_F52del*^) proteins. **A-R.** Embryonic phenotypes. Images of wild-type larvae at 96-hpf (A-E) and 120-hpf (F), as well as embryos carrying the p.(Gln48Serfs*5) frameshift (G-L) or p.(Arg51_Phe52del) in-frame deletion (M-R) alleles are shown. Whole mount images (A-C, G-I, and M-O), as well as frontal (E, J, K, Q) and sagittal (D, P) ocular sections are presented. Please notice reduced eye size (G-K), coloboma (white arrows in H), degenerative (H-J) or absent (K) lens, disorganized retina and irregular cornea (J, K) in embryos with frameshift mutations, as well as severe coloboma (white arrows in N) with disorganized retina, discontinuous RPE (white arrows in P, Q), and corneal defects (O) but overall comparable to wild-type eye size and lenses (M-Q) in embryos that are homozygous for the in-frame deletion. Alcian blue stain of wild-type (F) and mutant embryos (L, R) identified defects in craniofacial development with primary defects in the development of the ceratohyal cartilage of the hyoid arch (or the second pharyngeal arch). **S-W.** Adult phenotype. Images of adult wild-type (S, T) and Arg51_Phe52del mutant (U-W) fish: please notice microphthalmic highly disorganized eye with pigmented cornea (V) and anophthalmic contralateral eye with residual abnormal pigmented tissue (W). Please also note normal appearance of pectoral fins in the mutant fish (U). ch, ceratohyal; ep, ethmoid plate; m, Meckel's cartilage; pq, palatoquadrate; e, eye; c, cornea; le, lens; ipl, inner plexiform layer; on, optic nerve; pf, pectoral fins; r, retina.

Zebrafish carrying heterozygous frameshift alleles were crossed to generate homozygous or compound heterozygous *mab21l2* mutants and heterozygous fish with the c.151_156delCGTTTC, p.(Arg51_Phe52del) in-frame deletion were bred to obtain homozygous embryos; the resulting embryos were examined to determine the associated phenotypes (see Fig. 4 for embryonic phenotypes associated with wild-type (A-F), frameshift (G-L) and in-frame deletion (M-R) alleles). Crosses involving frameshift mutations resulted in a distinct phenotype in 26 out of 101 (26%) embryos, consistent with a recessive mode of inheritance. The phenotype of homozygous frameshift mutants included microphthalmia with small or absent lens in 100% of affected embryos, as well as coloboma and shortened body/curved tail in 76% and 56% of affected fish, respectively (Fig. 4G-I, S2 Fig.). The phenotype was first evident in 24–48-hpf embryos and became progressively worse over time. All 26 abnormal fish from these crosses were found to either be compound heterozygous for the c.150_156delCCGTTTC, p.(Arg51Serfs*4) and c.151dupC, p.(Arg51Profs*14) alleles (9 embryos), homozygous for the c.155delT, p.(Phe52Serfs*5) allele (3), homozygous for the c.150_156delCCGTTTC, p.(Arg51Serfs*4) allele (2), homozygous for c.141_153delCCAAGAGCCCCGT p.(Gln48Serfs*5) mutation (9) or homozygous for the c.151delC, p.(Arg51Valfs*6) allele (3) by restriction digest and/or Sanger sequencing. Crosses involving heterozygous carriers of the c.151_156delCGTTTC, p.(Arg51_Phe52del) allele produced 12 out of 57 (21%) abnormal embryos, also consistent with a recessive mode of inheritance. The abnormal phenotype was first evident at 72-hpf and involved severe ocular coloboma in all affected embryos and corneal defects in 42% (5/12), while lenses and eye size appeared to be only mildly affected (Fig. 4M-O).

Mutant lines were established for the c.141_153delCCAAGAGCCCCGT, p.(Gln48Serfs*5) frameshift mutation (*mab21l2* ^Q48Sfs*5^) and the c.151_156delCGTTTC, p.(Arg51_Phe52del) in-frame deletion (*mab21l2* ^*R51_F52del*^) alleles (Fig. 3L and S1 Fig.) and were further characterized. Expression of mutant *mab21l2* transcripts was examined by RT-PCR using RNA extracted from 48-hpf embryos. Both mutant transcripts were found to be expressed at slightly higher levels than wild-type transcript, but this increase was not statistically significant (Fig. 3L, S1 Fig.).

Histological analysis of the 96–120-hpf homozygous embryos with *mab21l2* ^Q48Sfs*5^ and other frameshift mutations demonstrated a small degenerative (Fig. 4J) or absent (Fig. 4K) lens, disorganized retina (particularly the inner plexiform layer), and irregular cornea (Fig. 4J, K). Histological analysis of the *mab21l2* ^*R51_F52del*^ homozygous embryos confirmed severe coloboma, abnormally formed retina (inner plexiform layer) and cornea, discontinuous RPE (retinal pigmented epithelium), as well as generally normal eye size and lens appearance (Fig. 4P, Q). Alcian blue staining identified craniofacial defects in some *mab21l2* ^Q48Sfs*5^ (3/7; 43%) and *mab21l2* ^*R51_F52del*^ (3/6; 50%) 120-hpf homozygous fish: the cartilage elements of the pharyngeal arches, particularly the ceratohyal cartilage of the hyoid arch (or the second pharyngeal arch), were malformed and displaced (Fig. 4L, R) while the ethmoid plate cartilage of the upper jaw appeared to be largely unaffected. Analysis of the pectoral fin did not identify any obvious malformations in *mab21l2* ^Q48Sfs*5^ or *mab21l2* ^*R51_F52del*^ 120-hpf embryos or *mab21l2* ^*R51_F52del*^ adult fish (Fig. 4U).

Homozygous *mab21l2* ^Q48Sfs*5^embryos demonstrated 100% lethality with no embryos surviving into adulthood while two out of fifteen (13%) *mab21l2* ^*R51_F52del*^ homozygous larvae developed into adults. Gross morphological examination of adult fish revealed a severe ocular phenotype with anophthalmia of one eye and microphthalmia of the contralateral eye (see Fig. 4S, T for wild-type and 4U-W for mutant fish phenotypes). In addition to small size, the ocular structures appeared to be highly disorganized with an expanded anterior segment and pigmented cornea (Fig. 4V).

### Comparative analysis of apoptosis, proliferation and ocular markers in wild-type and *mab21l2* embryos

**Fig 5:**
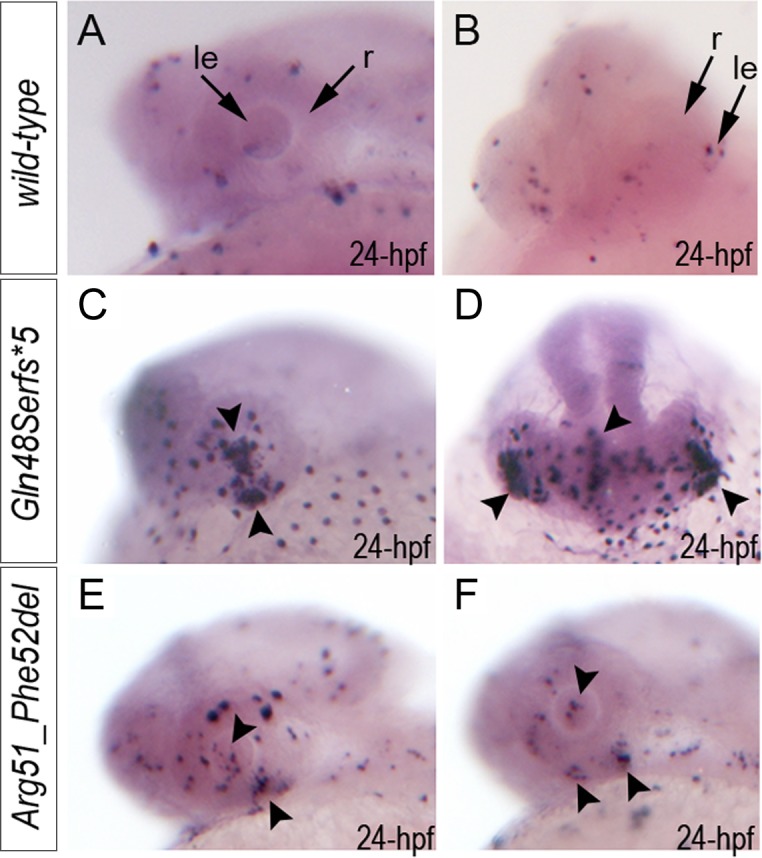
Summary of TUNEL assays in zebrafish wild-type and *mab21l2* mutant embryos. TUNEL results in 24-hpf wild-type (A-B), *mab21l2* ^Q48Sfs*5^ embryos (C-D) and *mab21l2* ^*R51_F52del*^ mutants (E-F) are shown. An increase in TUNEL staining was observed in both *mab21l2* mutants with remarkably high levels in the *mab21l2* ^Q48Sfs*5^ embryos, particularly in the lens and ventral retina (C-D), and moderately increased levels in the *mab21l2* ^*R51_F52del*^ embryos (E-F); arrowheads indicate sites of increased TUNEL staining in the eye and brain; le, lens; r, retina.

Analysis of apoptosis with TUNEL assay (that detects terminal deoxynucleotidyl transferase dUTP nick end labeling) identified an excessive number of TUNEL-positive cells in 24–72-hpf homozygous *mab21l2* ^Q48Sfs*5^ embryos in comparison to wild-type (Fig. 5A-D; S3 Fig.): at 24- hpf, the increased number of TUNEL-positive cells was observed in the developing lens and ventral retina (Fig. 5C, D); at 48–72-hpf clusters of TUNEL-positive cells continued to be found in lens, margins of the open optic fissure and other regions of the retina (possibly the outer nuclear layer) (S3 Fig.). In *mab21l2* ^*R51_F52del*^ mutants carrying the in-frame deletion allele, a moderate increase in TUNEL-positive cells was observed: at 24-hpf an increase in TUNEL-positive cells was detected in the ventral region of the retina with some staining in other retinal regions and in the lens (Fig. 5E, F); at 48–72-hpf, TUNEL staining continued to be seen primarily the retina and not in the lens (S3 Fig.). A moderate increase in TUNEL staining was also observed in the brain and other embryonic tissues in both mutants (Fig. 5D, S3 Fig.).

**Fig 6:**
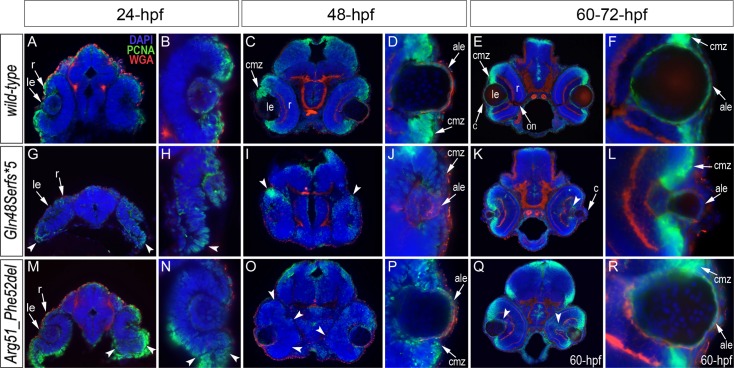
Analysis of proliferation patterns and differentiation markers in wild-type and mutant embryos. Immunostaining with PCNA (Proliferating Cell Nuclear Antigen) (green), DAPI (blue), wheat germ agglutinin (WGA) (red) was performed using 24–72-hpf wild-type embryos (A-F) as well as *mab21l2* ^Q48Sfs*5^ (G-L) and *mab21l2* ^*R51_F52del*^ (M-R) mutant tissues. Embryonic stages are indicated above; overlay fluorescence images are shown and single immunoreactivity data is available in S4 Fig. White arrowheads point to defects in retinal invagination and regions of abnormal retinal folding (H,M,N) observed in both *mab21l2* ^Q48Sfs*5^ and *mab21l2* ^*R51_F52del*^ embryos (please see text), as well as areas of aberrant PCNA labeling (I,K,O,Q). Please also note the absence of PCNA staining in the anterior lens epithelium of 48- and 72-hpf *mab21l2* ^Q48Sfs*5^ mutants (J,L). ale, anterior lens epithelium; c, cornea; cmz, ciliary marginal zone; le, lens; on, optic nerve; r, retina.

Immunohistochemistry with PCNA (Proliferating Cell Nuclear Antigen) was performed to compare proliferation patterns in 24–72-hpf wild-type and both *mab21l2* ^Q48Sfs*5^ and *mab21l2* ^*R51_F52del*^ mutant embryos; the monoclonal antibody ZL-1 was used to examine zebrafish fiber cell differentiation and the sections were counterstained with wheat germ agglutinin (WGA) and/or DAPI and to visualize cell membranes and nuclei, correspondingly (Fig. 6 and S4 and S5 Fig.). Strong nuclear PCNA immunoreactivity indicating robust proliferation was observed in the retinal marginal zone in 24-, 48- and 72-hpf wild-type embryos (Fig. 6A-F) as well as the developing lens, particularly in the anterior lens epithelium at 48- and 72-hpf (Fig. 6D, F). In *mab21l2* ^Q48Sfs*5^ mutant embryos, the intensity of PCNA staining appears to be diminished at early stages (Fig. 6G, H) with a disorganized pattern being observed in the retina at 48–72-hpf (Fig. 6I-L); no proliferation activity was detected in the anterior lens epithelium of either 48- or 72-hpf embryos (Fig. 6J and L) in these mutants. In the *mab21l2* ^*R51_F52del*^ deletion mutant, strong PCNA staining in the peripheral parts of the developing retina was observed at 24-hpf (Fig. 6M, N); disordered patterns were evident in 48- and 60-hpf embryos (Fig. 6O-R) with patches of PCNA-positive cells seen in the central regions of the retina (arrowheads in Fig. 6Q); in contrast to the frameshift mutant, PCNA staining was observed in the cells of anterior lens epithelium in both 48- and 60-hpf *mab21l2* ^*R51_F52del*^ embryos (Fig. 6P, R). ZL-1 positive cells were observed in wild-type embryos starting from 24-hpf (S5 Fig.); this staining was not noted in the abnormal eyes of *mab21l2* ^Q48Sfs*5^ mutants at 24-hpf but was detected in 48- and 72-hpf *mab21l2* ^Q48Sfs*5^ embryos and 72-hpf *mab21l2* ^*R51_F52del*^ mutants (S5 Fig.).

Both mutants demonstrated abnormal retinal shape at 24-hpf (Fig. 6G, H, M, N) suggesting a possible invagination defect; a shallow optic cup, particularly at its ventral region, was detected in the *mab21l2* ^Q48Sfs*5^ frameshift mutants (Fig. 6G, H) and a smaller, more constricted, optic vesicle was present in the *mab21l2* ^*R51_F52del*^ deletion mutants (Fig. 6M, N); abnormal retinal folding was evident in both mutants at 24- and 48-hpf (arrowheads in Fig. 6G, H, M-O).

**Fig 7:**
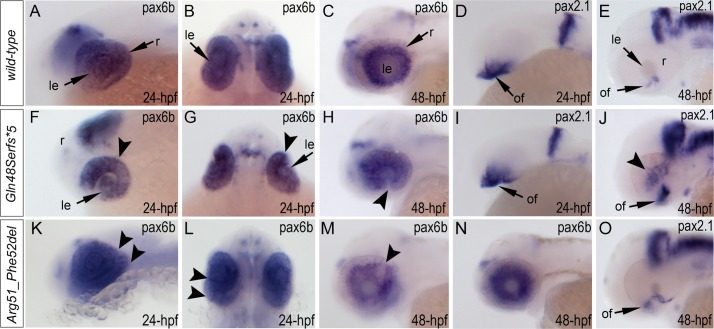
Analysis of *pax6b*, *pax2*.*1* and *foxe3* expression in wild-type, *mab21l2* ^Q48Sfs*5^ and *mab21l2* ^*R51_F52del*^ embryos. Wild-type (A-E) and mutant (F-O) zebrafish embryos at 24–48-hpf were analyzed as indicated in the right bottom corner of each image. Please note a change in *pax6b* transcript distribution at 24-hpf and 48-hpf in *mab21l2* ^Q48Sfs*5^ (arrowheads in F-H), retinal folding defect in *mab21l2* ^*R51_F52del*^ embryos at 24-hpf (arrowheads in K, L) and visibly abnormal *pax6b* pattern at 48-hpf in some (arrowheads in M) but not all (N) *mab21l2* ^*R51_F52del*^ embryos. *pax2*.*1* expression seems to be unaffected in 24-hpf frameshift mutant embryos (I) but shows an abnormal pattern in both mutants at 48-hpf (J,O). At 48-hpf, in addition to more broad and intense *pax2*.*1* expression in the region of optic fissure, abnormal *pax2*.*1* staining was detected in central retina in *mab21l2* ^Q48Sfs*5^ embryos (arrowheads in J). le, lens; of, optic fissure; retina.

Expression of the *pax6b*, *pax2*.*1* and *foxe3* genes was examined in wild-type and mutant fish (Fig. 7, S5 Fig.). Expression of *pax6b* in wild-type embryos was detected throughout the developing eye at 24-hpf (Fig. 7A, B) with a more restricted pattern in the retinal ganglion cells and inner nuclear layer neurons at 48-hpf (Fig. 7C). In *mab21l2* ^Q48Sfs*5^ mutants, *pax6b* expression was detected in both the developing lens and retina but appeared to be downregulated in the ventral/temporal versus dorsal/nasal domain (arrowhead in Fig. 7F, G); at 48-hpf *pax6b* expression was not present in the region of the coloboma (arrowhead in Fig. 7H). In the *mab21l2* ^*R51_F52del*^ mutants, *pax6b* expression was detected throughout the abnormally folded retina at 24-hpf (Fig. 7K, L); at 48-hpf, 50% (2/4) of the embryos showed patchy pattern of *pax6b* expression (Fig. 7M) while others (2/4) demonstrated a grossly normal *pax6b* distribution (Fig. 7N). We also examined zebrafish *pax2*.*1* expression, which normally shows robust staining at the opposite edges of the open fissure at early embryonic stages (24-hpf) (Fig. 7D) and becomes diminished and more restricted to the optic nerve and the site of choroid fissure as the fissure begins to close (by 48-hpf) (Fig. 7E, S5 Fig.). In *mab21l2* ^Q48Sfs*5^ homozygous mutants, *pax2*.*1* expression appears to be unaffected at 24-hpf (Fig. 7I) but an altered pattern is observed at later stages with broad and intense expression continuing in the region of optic fissure (Fig. 7J, S5 Fig.) and abnormal areas of *pax2*.*1*-positive cells being noticeable in the central retina (arrowheads in Fig. 7J and S5 Fig.). In the *mab21l2* ^*R51_F52del*^ mutants, *pax2*.*1* expression is expanded in 48-hpf embryos and continues to be observed at the edges of the retina at the site of coloboma at 72-hpf (Fig. 7O, S5 Fig.); no *pax2*.*1* positive patches were detected in other retinal regions. Expression of *foxe3* (which marks the lens epithelial layer) in the developing 48-hpf lens seemed to be somewhat downregulated in both mutants with mutant lenses also appearing smaller than wild-type lenses (S5 Fig.).

### Analysis of wild-type and mutant MAB21L2/mab21l2 proteins

**Fig 8:**
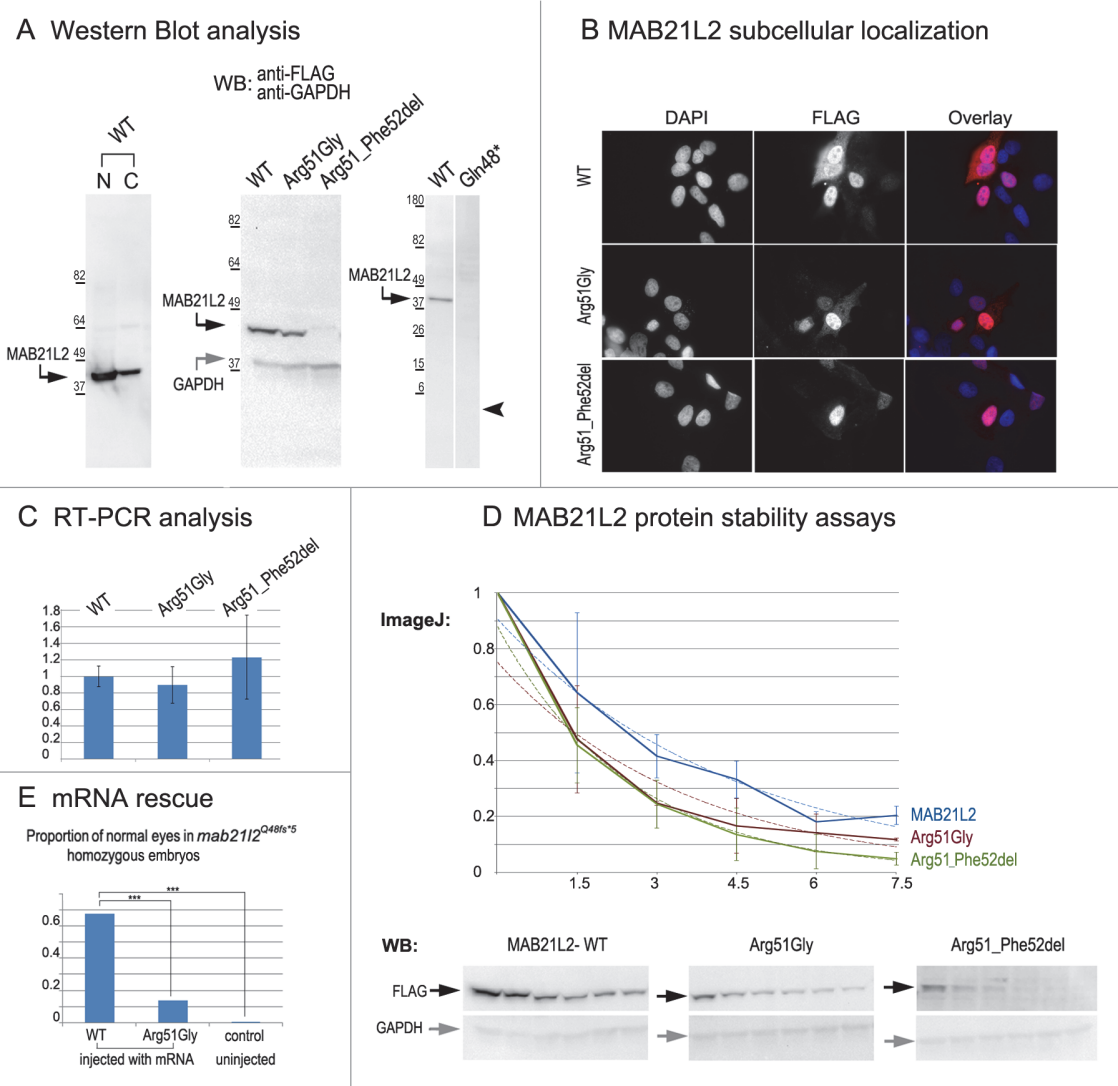
MAB21L2 wild-type and mutant protein studies. **A.** Western Blot analyses of MAB21L2 wild-type and mutant proteins (with N-terminal FLAG tag) in HLE-B3 cells. Please note the presence of wild-type MAB21L2 in both nuclear (N) and cytoplasmic (C) fractions (left), decreased expression of Arg51Gly and Arg51_Phe52del mutants (center) and the absence of Gln48* mutant protein (right; arrowhead indicates region corresponding to 5.5kDa, the predicted molecular weight for this peptide); predicted molecular weight for MAB21L2 is ~41kDa and for GAPDH is ~36 kDa; positions and corresponding molecular weights of protein ladder are shown for every blot. **B.** Cellular immunofluorescence analysis demonstrated no significant alteration in the localization pattern between wild-type and mutant (Arg51Gly and Arg51_52del) proteins with predominant nuclear localization and some cytoplasmic staining for all forms. **C.** RT-PCR analysis of MAB21L2 wild-type and mutant transcripts in human lens epithelial cells transfected with corresponding expression constructs. **D.** Protein stability assays with cycloheximide showed a more rapid decrease in the amount of Arg51Gly and Arg51_Phe52del mutant proteins in comparison to wild-type protein. Western blots signals were measured using ImageJ software, and obtained values were graphed to produce degradation curves represented by solid blue (wild-type), red (Arg51Gly) and green (Arg51_Phe52del) lines (standard deviations for every time point are indicated as thin vertical lines and exponential decay curves fitted into each graph are shown as dotted lines of corresponding colors); representative Western blot (WB) images are shown on the bottom (stability assays were performed in triplicate; 0, 1.5, 3, 4.5, 6, and 7.5 correspond to hours of exposure to cycloheximide). **E.** Summary of mRNA rescue experiments. Proportions of embryos with normal eyes in homozygous mab21l2Q48fs*5 embryos injected with wild-type human *MAB21L2* mRNA, mRNA encoding the p.(Arg51Gly) mutant protein, and uninjected control larvae are shown; statistically significant (p<0.0005) differences are indicated with asterisks (***).

To examine the effect of the p.(Arg51Gly) mutation on the MAB21L2 protein, we tested the expression, localization and protein stability of the wild-type and mutant proteins via transfections of the corresponding FLAG-tagged constructs into human lens epithelial B3 (HLE-B3) cells [32]; *MAB21L2* was also found to be endogenously expressed in these cells (S1 Fig.). Mab21l proteins were previously shown to localize primarily to the nucleus [33]. Western Blot analysis of nuclear and cytoplasmic fractions confirmed the presence of ~41kDa wild-type MAB21L2 protein in both cellular compartments with greater nuclear localization (Fig. 8A). Western blot analysis of whole-cell extracts detected a decreased amount of the p.(Arg51Gly) mutant in comparison to wild-type protein (Fig. 8A); the mutant protein was found to be present at 31.97% ± 9.36% of wild-type based on three independent experiments. Cellular immunofluorescence analysis demonstrated no significant alteration in the localization pattern between wild-type and mutant protein with predominant nuclear localization and some cytoplasmic staining for both forms (Fig. 8B). Since the levels of recombinant *MAB21L2* transcript encoding for wild-type and p.(Arg51Gly) mutant proteins detected by RT-PCR did not show a significant difference (Fig. 8C, S1 Fig.), we proceeded to perform protein stability assays using a series of cycloheximide treatments of HLE-B3 cells transfected with MAB21L2 constructs (Fig. 8D). Protein stability assays showed a more rapid decrease in the amount of p.(Arg51Gly) mutant in comparison to wild-type protein at all examined time points, with 11% of the mutant protein being present after 7.5 hours of cycloheximide treatment in comparison to 20% of the wild-type MAB21L2 (Fig. 8D). Calculation of the proteins’ half-life identified a statistically significant difference (p<0.05) in stability of wild-type (2.64±0.25 hours) versus the p.(Arg51Gly) mutant (1.91±0.36). These data suggest that the substitution of the arginine at position 51 to glycine affects MAB21L2 stability.

Functional analysis of the p.(Gln48*) (to represent the zebrafish p.(Gln48Serfs*5) mutant) and p.(Arg51_Phe52del) proteins was also undertaken in HLE-B3 cells. The p.(Gln48*) mutant was found to be highly unstable as no detectable level of this protein (estimated to have a molecular weight of ~5.5 kDa) was identified via Western blot analysis (Fig. 8A). The p.(Arg51_Phe52del) mutant also demonstrated a reduction in the amount of detected protein to 2.57% ± 2.20% of wild-type MAB21L2 protein based on three independent experiments (Fig. 8A); cellular immunofluorescence analysis demonstrated predominantly nuclear staining similar to wild-type distribution (Fig. 8B). Protein stability assays confirmed increased instability of the p.(Arg51_Phe52del) protein with less than 5% of mutant protein being detected after 7.5 hours of cycloheximide exposure versus 20% for the wild-type (Fig. 8D). The half-life of the p.(Arg51_Phe52del) mutant was estimated to be 1.91±0.27 hours versus 2.64±.25 for wild-type, which represented a statistically significant difference (p<0.05). The half-life values of the p.(Arg51_Phe52del) and p.(Arg51Gly) proteins were not significantly different from each other despite the observed reduced level of p.(Arg51_Phe52del) in comparison to p.(Arg51Gly) in replicate Western blots of untreated cells (see above, Fig. 8A); this suggests that there may be additional factors affecting the stability of the p.(Arg51_Phe52del) mutant which may require alternative assays to uncover [34].

To further test the pathogenicity of the human p.(Arg51Gly) allele, we injected embryos generated by heterozygous *mab21l2* ^Q48Sfs*5^ crosses with either wild type *MAB21L2* mRNA or mRNA encoding for the p.(Arg51Gly) mutant; a small number of embryos were left uninjected as a control. The embryos were carefully examined for ocular anomalies at 72-hpf, divided into affected and normal groups, and then genotyped. In total, out of 104 surviving embryos injected with wild type *MAB21L2*, 68% (21/31) of homozygous fish developed normal eyes and lenses while out of 108 surviving embryos injected with the mutant mRNA encoding for p.(Arg51Gly), only 14% (3/22) of homozygous fish developed normal eyes and lenses; in the control group, none of the 9 uninjected homozygous embryos (out of 36 surviving) had normal eyes and lenses. The difference in the proportion of normal phenotypes seen in the homozygous *mab21l2* ^Q48Sfs*5^ fish injected with wild-type mRNA versus embryos injected with either mutant mRNA or left uninjected was statistically significant (p<.0005), while the difference between the mutant RNA- injected and the uninjected embryos was not statistically significant (p>.20) by chi-squared analysis (Fig. 8E). The efficient rescue by wild type *MAB21L2* mRNA confirms that the ocular phenotype in the mutant line is caused by the *mab21l2* deficiency and demonstrates functional conservation between zebrafish and human mab21l2/MAB21L2 proteins. The absence of robust rescue by mRNA encoding for the p.(Arg51Gly) mutant supports the pathogenic role of this *MAB21L2* allele in human disease.

To investigate the possibility of dominant-negative effects for the mutant allele, *MAB21L2* wild type mRNA or mRNA encoding for the p.(Arg51Gly) mutant were injected (at the same concentrations as in rescue experiments) into wild-type embryos and observed for phenotypes up to 120-hpf. Among the 39 surviving embryos that were injected with wild-type *MAB21L2*, no embryos displayed an eye phenotype (9 fish showed moderate-severe overall malformations). Similarly, among the 61 surviving embryos that were injected with the mutant mRNA encoding for the p.(Arg51Gly) protein, no embryos showed an eye phenotype (10 fish showed moderate-severe overall malformations). Thus, these experiments identified no dominant-negative effect for the p.(Arg51Gly) mutant allele in zebrafish at concentrations that are sufficient for phenotypic rescue of the *mab21l2*-deficient phenotype.

## Discussion

In this manuscript we present evidence for the conserved function of *MAB21L2/mab21l2* in vertebrate ocular development through demonstration of abnormal phenotypes associated with mutations in this gene in humans and zebrafish. Human *MAB21L2*, located at 4q31.3, is a member of the *MAB21L* gene family that consists of three members, *MAB21L1-3*. MAB21L proteins are related to the mab-21 (male abnormal 21) nuclear factor [33] associated with thinning of ray 6 and frequent fusions of rays 4 and 6 of a set of nine bilateral peripheral rays in male *C*. *elegans* [27]. The molecular function of MAB21L2 is largely unknown. Previous reports involving double mutants of *mab-21* and *sma* members (*SMAD* family) in *C*. *elegans* indicated that *mab-21* is positioned downstream of *sma* and is negatively regulated by *dbl-1*, a homolog of human *BMP5*, thus placing *mab-21* in the TGF-β signaling cascade [35,36]. The TGF-β signaling cascade plays an important role in ocular development and mutations in many associated factors, such as *BMP4*, *BMP7*, *GDF3*, and *GDF6*, lead to an overlapping spectrum of phenotypes in humans [13,14, 37–39]. Additionally, mab-18 (PAX6 ortholog) mutants demonstrate a phenotype that is highly similar to mab-21 *C*. *elegans* mutants [27] and *Mab21l2* expression was found to be upregulated in embryonic mouse lenses heterozygous for a *Pax6* loss-of-function allele [40] suggesting a possible conserved genetic interaction.

In mouse, *Mab21l2* ocular expression was reported in the dorsal optic vesicle and head surface ectoderm in E8.5–9.5 embryos and the neural retina, optic nerve and RPE at E12 [41,42]; homozygous *Mab21l2* knockout mice demonstrated a rudimentary retina and aphakia due to improper invagination of the optic vesicle as well as ventral body wall defects, improper formation of the heart and liver, and embryonic lethality [41,42]. Previously published reports of lens expression in animal models are somewhat inconsistent: four studies reported lack of lens expression for *Mab21l2/mab21l2* (with many showing the presence of lens expression for its close homolog, *Mab21l1/ mab21l1*) [41,43–45] while three other manuscripts noted *Mab21l2/mab21l2* transcripts in the developing lens [33,46,47], which is consistent with the results of our study. In zebrafish, morpholino-mediated knockdown of *mab21l2* resulted in microphthalmia and incomplete retinal development including discontinuous inner and outer plexiform layers [47]. In the zebrafish genetic mutants that we developed, severe defects in the development of both the lens and retina were observed consistent with strong expression of *mab21l2* in both tissues. While the phenotypes of *mab21l2* morphants and mutants show overlap, some ocular features, including severe lens degeneration, coloboma, and early retinal invagination defects, appear to be more pronounced or observed primarily in genetic mutants; this may be due to incomplete disruption of mab21l2 function via morpholino, the transient nature of morpholino-induced effects, and/or genetic background differences.

Both of the *mab21l2* mutants generated in this study demonstrated variable retinal invagination defects. In zebrafish, retinal invagination, which occurs upon contact of the optic vesicle (formed as an extension of the forebrain neuroepithelium) with the surface ectoderm, is completed with formation of the cup-shaped optic vesicle by ~22-hpf. In *mab21l2* ^Q48Sfs*5^ embryos, a shallow misshapen optic cup, especially at its ventral part, was detected in 24-hpf embryos; the lens mass (similar to the mammalian lens vesicle) was also visibly smaller than normal at this stage. In the *mab21l2* ^*R51_F52del*^ in-frame deletion mutant, a more constricted optic cup with some ventral and dorsal retinal folding was observed, while the developing lens appeared to have normal size and shape in most embryos. The observed defects in early optic cup formation indicate a possible conserved role for *mab21l2* in this process in vertebrates.

Studies of proliferation and cell death in zebrafish wild-type and *mab21l2* ^Q48Sfs*5^ or *mab21l2* ^*R51_F52del*^ homozygous mutant embryos identified multiple deviations from normal patterns. The most remarkable finding was a significant increase in TUNEL staining in the developing retina and lens of *mab21l2* ^Q48Sfs*5^ mutants at early embryonic stages. An increase in TUNEL staining was also observed at later stages of development and was, to a lesser degree, present in the *mab21l2* ^*R51_F52del*^ embryos carrying homozygous in-frame deletion alleles. Both increased cell death (through apoptosis and/or necroptosis) and defects in cellular proliferation have been linked to optic fissure closure defects in other genetic models of coloboma [48–59]; since the TUNEL assay may not discriminate between apoptotic and other cell death mechanisms [60], further studies are needed to evaluate the possible involvement of necroptosis in *mab21l2* deficient phenotypes. Additionally, the expression of several ocular markers including *pax6b*, *pax2*.*1*, *foxe3* and ZL-1 was altered in *mab21l2* mutants. These observed differences seem more likely to be caused by the severe disorganization of mutant ocular tissues rather than direct regulation of expression of those markers by mab21l2.

The distinct phenotypes associated with the *mab21l2* ^Q48Sfs*5^ frameshift truncation and *mab21l2* ^*R51_F52del*^ in-frame deletion mutations suggest different molecular mechanisms. The *mab21l2* ^*R51_F52del*^ in-frame deletion demonstrated milder features in comparison to the *mab21l2* ^Q48Sfs*5^ mutant, such as less affected lens development, less pronounced cell death and incomplete embryonic lethality. These observations support the possibility that this mutation results in a hypomorphic allele retaining some of its normal function, particularly in relation to lens development. The severe coloboma observed in *mab21l2* ^*R51_F52del*^ embryos may point to the particular importance of domains located in the N-terminal region of mab21l2 for its proper functioning in the developing retina. Since the zebrafish p.(Arg51_Phe52del) in-frame deletion and the human p.(Arg51Gly) missense alleles affect homologous regions of the MAB21L2/mab21l2 proteins, the mechanism(s) of these mutations may be similar to each other. Understanding of these mechanisms requires additional studies into the domain structure and function of MAB21L2/mab21l2 protein.

Analysis of wild-type and mutant proteins in human lens epithelial cells identified a range of protein stability defects among mutant forms with the human p.(Arg51Gly) mutant stability mildly reduced, the zebrafish p.(Arg51_Phe52del) mutant stability highly affected, and the p.(Gln48*) truncation protein (similar to the p.(Gln48Serfs*5) mutant) being entirely unstable. This analysis suggested that the zebrafish p.(Gln48Serfs*5) mutation results in a complete loss-of-function allele, since the similar mutant protein p.(Gln48*) was completely unstable and any protein produced would be missing the entire mab-21 domain. These experiments also indicated the likely importance of arginine 51 and the immediately adjacent amino acids for normal conformation and stability of the MAB21L2/mab21l2 protein because disruptions of this region resulted in decreased stability for both the human p.(Arg51Gly) and the zebrafish p.(Arg51_Phe52del) proteins. Further studies demonstrated that, in contrast to wild-type human *MAB21L2* mRNA, mutant mRNA encoding for the p.(Arg51Gly) protein failed to efficiently rescue the ocular phenotype of homozygous *mab21l2* ^Q48Sfs*5^ embryos, suggesting functional deficiency for the identified human mutation. In addition, overexpression of mutant *MAB21L2* mRNA in zebrafish embryos resulted in no obvious ocular phenotype, implying absence of a dominant-negative effect. However, it is possible that the full impact of the p.(Arg51Gly) mutation was not discernable due to insufficient dosage, its acting later in development, or other factors; this needs to be further investigated by additional approaches, including development of zebrafish mutants carrying this specific allele.

No abnormal phenotype was seen in heterozygous zebrafish for either the frameshift truncation or in-frame deletion mutations, while a dominant phenotype was observed in human patients with the p.(Arg51Gly) alteration. This may be explained by variability in gene dosage requirements between species [61] or a different mechanism for the human mutation. The presence of mild rhizomelia/ contractures in human patients with *MAB21L2* mutations suggests its involvement in skeletal development. Additional phenotypes such as shortened body/curved tail, craniofacial malformations and lethality were present in the zebrafish *mab21l2* mutants while heart, liver, embryonic lethality and other anomalies were previously reported in mouse *Mab21l2* mutants [41,42]. Considering these observations and the fact that multiple pleiotropic effects of mab-21 mutations have been previously noted [27], additional *MAB21L2*-associated phenotypes are likely to be identified and may present with broad interfamilial variability.

While this paper was in revision, four families with mutations in *MAB21L2* were described by Rainger et al [62]. This manuscript reports three heterozygous mutations (p.(Arg51Cys), p.(Arg51His), and p.(Glu49Lys) affecting the same region as the mutation reported here and resulting in similar phenotypes (anophthalmia or colobomatous microphthalmia with rhizomelic skeletal dysplasia in two families); one of the reported families includes the UK10K_COL5001067 and UK10K_COL5001068 cases with the p.(Arg51His) mutation that we presented in results from the publically available data. In addition, one family with a recessive *MAB21L2* mutation affecting the C-terminal region of the protein was identified. Functional assays of the mutant alleles using an inducible HEK293 cell system expressing wild-type or mutant MAB21L2 as GFP fusion proteins suggested a possible gain-of-function effect for the dominant mutations based on observed increased protein stability; an increase in phospho-ERK1 was detected with induction of either wild-type or p.(Arg51His) mutant MAB21L2 protein [62]. This differs from the proposed mechanism of the mutation identified in our study which revealed a decrease in protein stability for the p.(Arg51Gly) mutant tagged with FLAG epitope and absence of a dominant phenotype in zebrafish embryos injected with mRNA encoding for this protein. This may be due to diverse effects of the studied amino acid substitutions (Arg51Gly versus Arg51His, Arg51Cys, and Glu49Lys) on MAB21L2 protein structure, different effects of tags on protein stability (GFP versus FLAG), or other factors, and needs to be investigated further. Rainger and co-authors (2014) also suggested an involvement of MAB21L2 in ssRNA binding [62]. In our manuscript, we developed an allelic series of zebrafish *mab21l2* mutants, uncovered an early onset optic cup apoptosis phenotype in affected embryos, and demonstrated a decrease in protein stability, as well as functional deficiency and no dominant-negative effect for the p.(Arg51Gly) mutation in zebrafish embryos.

Taken as a whole, the complete co-segregation of the identified *MAB21L2* allele with the ocular phenotype in the family, the *de novo* appearance of the mutation in the affected progeny of unaffected parents with low-level mosaicism present in the mother, the in silico and experimental indications of the functional importance of the arginine at position 51, the development of ocular defects in *mab21l2* mutant zebrafish embryos, as well as rescue of the ocular phenotype of homozygous *mab21l2* ^Q48Sfs*5^ embryos with injection of wild-type but not the p.(Arg51Gly)-encoding mutant mRNA, provide strong support for the pathogenicity of the identified *MAB21L2* allele and the involvement of *MAB21L2* in human ocular development and disease. The developed zebrafish *mab21l2* mutants will help to further explore the developmental roles and molecular function of this conserved ocular factor.

## Materials and Methods

### Ethics statement

The human study was approved by the Children’s Hospital of Wisconsin Institutional Review Board (protocol number CHW 03/56) with written informed consent obtained from each participant and/or their legal representative, as appropriate. This study also utilized data generated by the UK10K Consortium, derived from samples from UK10K_Rare_Coloboma (EGA Study ID: EGAS00001000127); a full list of the investigators who contributed to the generation of the data is available from http://www.UK10K.org. Access to data generated by the UK10K_Rare_Coloboma (EGA Study ID: EGAS00001000127) project of the UK10K Consortium study (http://www.UK10K.org) was obtained through a Data Access Agreement. The animal protocol was approved by the Institutional Animal Care and Use Committee at the Medical College of Wisconsin (protocol number AUA00000351_AR_5).

### Human sequencing studies

Whole exome sequencing of DNA samples of the proband’s two affected nephews was undertaken. Library capture was completed using the Agilent Sure Select v4 capture kit (Santa Clara, CA) and 100 base pair paired end sequencing was performed using the Illumina HiSeq 2000 at Axeq Technologies (Rockville, MD). The obtained data were aligned using the Burrows-Wheeler Aligner (BWA) and variants were called using the SAMTOOLS analysis pipeline available through Axeq. Exome data were analyzed using the SNP & Variation Suite (Golden Helix, Bozeman, MT) to identify variants that are shared between the affected individuals and then prioritized based on their absence/rarity in the general population (as reported in publicly available databases dbSNP (http://www.ncbi.nlm.nih.gov/snp), NHLBI Exome Sequencing Project Exome Variant Server (EVS; http://evs.gs.washington.edu/EVS/), and 1000 Genomes (http://www.1000genomes.org/data), as well as our own data) and possible effect on protein function; for missense variants, functional profiling was performed using SIFT, Polyphen2, Mutation Taster, MutationAssessor, and FATHMM as well as nucleotide conservation scores GERP++ and PhyloP as accessed through dbNSFP [63].

To confirm the identified *MAB21L2* changes and perform cosegregation analysis, DNA from all available family members was amplified using the primers and conditions provided in S2 Table. PCR products were sequenced bidirectionally using Big Dye Terminator chemistry and the ABI 3730XL sequencer (Applied Biosystems/Life Technologies, Carlsbad, CA, USA). Sequences were reviewed manually and using Mutation Surveyor (SoftGenetics, State College, PA) and compared to the NM_006439.4 transcript.

Variant Call Format (VCF) files from the UK10K_Rare_Coloboma (EGA Study ID: EGAS00001000127) project of the UK10K Consortium study (http://www.UK10K.org) were analyzed using the SNP & Variation Suite (Golden Helix, Bozeman, MT) with filtering for the *MAB21L2* gene only. DNA samples from this cohort were not available for independent confirmation of the identified variants.

Additional human samples were screened using Sanger sequencing of the 1362-bp PCR product encompassing the full *MAB21L2* coding region using PCR primers and two extra internal primers (S2 Table). Sequences were analyzed by manual inspection and Mutation Surveyor software as described above.

### MAB21L2 protein studies

Protein sequences were aligned using the Kalign multiple sequence alignment tool (http://www.ebi.ac.uk/Tools/msa/kalign). The accession numbers for the MAB21L sequences used in the protein alignment are as follows: human MAB21L2 (AF155219.1), murine Mab21l2 (AF223425.1), zebrafish mab21l2 (AY038031.1), human MAB21L1 (NM_005584), murine Mab21l1 (AF228913.1), zebrafish mab21l1 (NM_152974.2), human MAB21L3 (NM_152367.2), murine Mab21l3 (NM_172295.4), zebrafish mab21l3 (NM_001110025.1), *Drosophila melanogaster* mab-21 (NP_651971.2), *Caenorhabditis elegans* mab-21 (NP_497940.2).

The expression construct for MAB21L2 wild-type protein with N-terminal FLAG (Cat. #EX-V1703-M11) was purchased from GeneCopoiea (Rockville, MD). The mutations were introduced using the Agilent QuikChange Lightning (Cat. #210519) site directed mutagenesis kit (Santa Clara, CA). Mutagenesis primers were designed using the online QuikChange Primer Design tool. HPLC purified mutagenesis primers were ordered from Thermo Fisher Scientific (Waltham, MA) and sequences were as follows: MAB21L2-p.R51G-Sense—5’-gaggtgcaggagcctggcttcatcagct-3’, MAB21L2-p.R51G-Antisense—5’-agctgatgaagccaggctcctgcacctc-3’, MAB21L2-p.51_52del-Sense—5’-gctcaaggagctgataggctcctgcacctc-3’, MAB21L2-p.51_52del-Antisense—5’- gaggtgcaggagcctatcagctccttgagc-3’, MAB21L2- Q48X-Sense—5’- gaagcgaggctcctacacctccacttcct-3’, MAB21L2- Q48X-Antisense—5’- aggaagtggaggtgtaggagcctcgcttc-3’.

Human lens epithelial B3 (HLE-B3) cells (ATCC, CRL-11421) were maintained in MEM (Life Technologies) supplemented with 20% FBS (Life Technologies), 2 mM glutamine (Life Technologies), and 1 mM sodium pyruvate (Life Technologies). All transfections were performed by transfecting HLE-B3 cells with 15 μg of DNA and 30 μl of Lipofectamine 2000 (Life Technologies) in Opti-MEM in 100 mm petri dishes.

For Western blot analysis, nuclear and cytoplasmic fractions were generated using the CelLytic NuCLEAR Extraction kit (Cat#NXTRACT Sigma-Aldrich,). 48 hours after transfection, cells were washed with PBS, collected, and resuspended in 0.5 ml of hypotonic lysis buffer and incubated on ice for 15 minutes, 30 μl of 10% IGEPAL was added to the cell suspension. Cells were then vortexed and spun at 10,000 xg for 30 seconds at 4°C. The supernatant (cytoplasmic fraction) was decanted and saved. The nuclear fraction was generated by adding 70 μl of nuclear extraction buffer to the pellet, incubating on ice for 25 minutes, spun at 20,000 xg for 5 minutes at 4°C and the supernatant was saved (nuclear fraction). Molecular weights for wild-type and mutant proteins were estimated using Protein Molecular Weight calculator (http://bioinformatics.org/sms/prot_mw.html). The BenchMark Pre-stained Protein Ladder (Cat. #10748-010) (Life Technologies) was utilized as the protein molecular weight standard.

The stability of the MAB21L2 wild type and mutant proteins were characterized using a modified protocol previously described by Alur et al. 2010 [64]. In these experiments, cycloheximide, which is known to block translational elongation and thus to inhibit protein biosynthesis in eukaryotic organisms, was used to treat cells transfected with MAB21L2 expression constructs in a time-course analysis to allow for protein degradation rates to be revealed in the absence of new protein production. Briefly, cells were split 24 hours after transfection into 35 mm petri dishes. After an additional 24 hours, cells were treated with 100 μg/ml cycloheximide (Cat. #C4859; Sigma-Aldrich) and collected at the indicated time points (0-, 1.5-, 3-, 4.5-, 6, and 7.5-hours after exposure). Whole cell lysates were generated by washing with PBS, cells were scraped in PBS and collected in 1.5 ml tubes, cells were spun at 1,000 xg for 1.5 minutes and PBS was removed. Cells were then resuspended in 40 μl of 1x RIPA buffer (50 mM Tris-HCL pH 8.0, 150 mM NaCL, 0.1% SDS, 0.5% sodium deoxycholate, 1% Triton X-100) and incubated on ice for 15 minutes, vortexing every 5 minutes. Cells were spun down for 10 minutes at 20,000 xg at 4°C and the supernatant was used for Western Blot analysis (see below). Western blots signals for MAB21L2 were quantified using ImageJ software and normalized to GAPDH levels (also measured by ImageJ) and the obtained values were graphed to produce degradation curves. Stability assays were performed in triplicate. Exponential decay curves were fitted to each replicate stability assay and the half-lives were determined. A two-tailed student's t-test was used to determine if the groups were different.

Lysates were denatured by adding equal volume of 2x sample buffer (65.8 mM Tris-HCl, pH 6.8, 26.3% (w/v) glycerol, 2.1% SDS, and 0.01% bromophenol blue) (Cat. #161-0737) (Bio-Rad, Hercules, CA) followed by boiling for 4 minutes at 95°C and run on a 10% Criterion Tris-HCl Gel (Cat. #345-1018) (Bio-Rad). Gels were immunobloted with anti-FLAG (Cat. #F1804) (Sigma-Aldrich) antibody at a 1:1000 dilution or anti-GAPDH (Cat#ab8245) (Abcam, Cambridge, MA) antibody at 1:5000 dilution. The secondary antibody was goat anti-mouse IgG HRP conjugate (Cat. #12-349) (Upstate Cell Signaling Solutions) at a 1:2500 dilution. The blots were developed using the Chemiluminescent Nucleic Acid Detection Module Kit (Thermo Fisher Scientific).

For cellular immunofluorescence experiments cells were split 24 hours after transfection and seeded onto coverslips. After 24 hours the cells were fixed with 4% paraformaldehyde for 15 minutes at room temperature, washed twice with PBS, permeabilized with 0.25% Triton X-100 in PBS, washed twice with PBS, blocked with 10% normal donkey serum in PBS for 30 minutes at 37°C, incubated with 1:1000 anti-FLAG antibody in 10% normal donkey serum in PBS for 2 hours at 37°C, washed three times with PBS, incubated in 1:1000 Alexa Flour 568 donkey anti-mouse IgG (Cat. #A10037) (Life Technologies), washed twice with PBS, incubated for 5 minutes at room temp with 1:1000 DAPI in PBS, washed twice with PBS and mounted on glass slides.

### Expression studies, histology, apoptosis assays and immunohistochemistry in zebrafish

Zebrafish (*Danio rerio*) maintenance and developmental staging were performed as previously described [65]. The protocol was approved by the Institutional Animal Care and Use Committee at the Medical College of Wisconsin. The expression of *mab21l2*, *pax6b*, *pax2* and *foxe3* was studied in 18–72-hpf zebrafish embryos using transcript-specific antisense riboprobes and previously described protocols [65]. For the *mab21l2* probe, a 698-bp fragment covering the sequence between nucleotides 788 and 1485 (GenBank# AY038031.1; primers provided in S2 Table) was utilized; clones for *pax6b* (AL915181) and *pax2*.*1* (AL906738) were purchased from Open Biosystems (Huntsville, Alabama) and linearized with EcoRv and BamHI, respectively; for the *foxe3* probe, a full length 1369-bp fragment covering the sequence between nucleotides 16 and 1384 (GenBank #BC163348.1) was utilized.

Gross morphological and histological analysis of zebrafish embryos was performed as previously described [65]. For apoptosis analysis, whole mount TUNEL assay as described by Eimon was followed [66]. For immunohistochemistry, immunohistochemical staining of zebrafish frozen sections was performed with DAPI (Cat. #62247) (Thermo Scientific), PCNA at 1:100 (Cat. #P8825) (Sigma-Aldrich), ZL-1 at 1:100 (ZIRC), and wheat germ agglutinin at 5 μg/ml (Cat. #W32464) (Life Technologies) following the previously published protocols [67–70].

### Generation of *mab21l2* mutant lines

TALENs were constructed following the Sanjana et al (2012) protocol [23]. Briefly, *mab21l2* TALENs were designed to target the region surrounding the nucleotide corresponding to the mutation site in human patients (left TALEN—5’-GAAGGAGGTGGAGGTCCAA-3’ and right TALEN—5’-CTATCTCGCTCAGGGAGCT-3’). Interruption of the *mab21l2* target sequence was predicted to affect the BanII restriction site (GRGCYC) located between the left and right TALENs; thus BanII digestion of the PCR-amplified genomic fragment involving this region was utilized to determine TALEN cutting efficiencies as well as to identify mutant embryos/adult fish and was followed by DNA sequencing for confirmation. Zebrafish embryos at the 1–4 cell stage were injected with TALEN RNA; initial analysis of injected larvae confirmed genome editing events and ~200 injected embryos were raised to adulthood to generate mosaic founders; these fish were then bred to produce embryos carrying germline *mab21l2* mutations that were raised to adulthood, genotyped using the primers provided in S2 Table, and bred to generate homozygous/compound heterozygous embryos.

### RNA isolation, RT-PCR and mRNA injections

To detect transcript levels in HLE-B3 cells, RNA from cells was isolated following transfection with expression plasmids containing sequences encoding for either wild type, or p.(Arg51Gly) or p.(Arg51_Phe52del) MAB21L2 alleles using TRIzol Reagent (Cat. #15596-026) (Life Technologies) 48-hours after transfection. Each sample was treated with DNase I (Cat. #18068) and reverse transcribed in triplicate using the SuperScript III Reverse Transcriptase system (Cat. #18080) (Life Technologies). Following reverse transcription the samples were treated with RNase H (Cat. #18021). Samples were amplified for *MAB21L2* using the following primers: MAB21L2-FLAG-F 5’- CAAAGACGATGACGACAAGG-3’ and MAB21L2-rt-R 5’- GGTAGAGCACCACCTCAAATTC-3’ (PCR product equal 305 bp). The following *GAPDH* primers were used as a loading control: GAPDH-F 5’- CCAAGGTCATCCATGACAACT-3’ and GAPDH-R 5’- GAGGCAGGGATGATGTTCTG-3’ (PCR product equal 148 bp). Endogeneous expression of *MAB21L2* transcript in the human lens epithelial cell line was detected with the following primers MAB21L2-endogenous-F, 5'-CCAGGTGGAAAACGAGAGTG-3' and MAB21L2-rt-R, 5’-GGTAGAGCACCACCTCAAATTC-3’ (PCR product equal 384 bp).

To detect *mab21l2* transcript levels in zebrafish RNA, three independent samples, each containing two embryos of either homozygous *mab21l2* ^Q48Sfs*5^ and *mab21l2* ^*R51_F52del*^ mutants or wild type, were isolated using the RNAqueous-Micro Total RNA Isolation Kit (Cat. #AM1931) (Life Technologies). Each sample was reverse transcribed as described previously. Samples were amplified for *mab21l2* using the following primers: *mab21l2*-zf-T-F 5’-TCTTTTCCTGGGAGTTGTGC-3’ and *mab21l2*-zf-T-R 5’- CCCCATCTGGTTCAGGTAAA-3’ (PCR product equal 350 bp). Because *MAB21L2/mab21l2* represents a single exon gene, a minus-reverse transcriptase control was included in each RT-PCR experiment to assure absence of contaminating DNA in each RNA sample. Amplification of the zebrafish gene for translation elongation factor 1 alpha (loading control) was performed with the following primers: *ef1a*x1-2F 5’- TCTCTCAATCTTGAAACTTATCAATCA-3’ and *ef1a*x3R 5’- AACACCCAGGCGTACTTGAA-3’ (PCR product equal 205 bp). The 1 Kb Plus DNA Ladder was utilized as nucleic acid standard (Cat. #10787-018) (Life Technologies). The PCR products were analyzed by electrophoresis and bands were quantitated using Image J software.

For rescue and overexpression experiments, mRNA was generated using the T7 mMESSAGE mMACHINE Transcription kit (Cat. #AM1344) (Life Technologies). 300 pg of mRNA was injected into 1–4 cell stage embryos produced by wild-type or c.141_153delCCAAGAGCCCCGT heterozygous mutant crosses. The injected embryos were observed for up to 5 days of fertilization, separated into groups based on observed presence/absence of ocular phenotype and then genotyped.

## Supporting Information

S1 Table Summary of novel or rare heterozygous variants predicted to be functionally significant. (DOC)

S2 Table PCR conditions and oligonucleotides utilized for amplification of gene regions in this study. (DOCX)

S1 Fig (A) DNA chromatograms that illustrate sequences of c.141_153delCCAAGAGCCCCGT, p.(Gln48Serfs*5) and c.151_156delCGTTTC, p.(Arg51_Phe52del) alleles (in heterozygous fish). (B) Agarose gel analysis of zebrafish mab21l2 transcript- specific amplification from 48-hpf homozygous zebrafish wild-type and mutant embryos; (**C**) Agarose gels representing RT-PCR expression analysis of recombinant *FLAG-MAB21L2* wild-type and mutant transcripts (left) as well as endogenous *MAB21L2* transcript in human lens epithelial cells (HLE-B3). RT minus (RT-) control refers to a mock reverse transcription containing all the RT-PCR reagents, except the reverse transcriptase, to demonstrate the absence of contaminating DNA in the corresponding RNA samples; *ef1a* and GAPDH amplification was performed for loading control in zebrafish and human samples correspondingly; the reactions were run on the same gel but irrelevant lanes were removed when needed; M- molecular marker, black arrow indicates *mab21l2-*, *FLAG-MAB21L2* or *MAB21L2*-specific bands of 350, 305 and 384 bp, correspondingly; black arrowhead points to ef1a- and GAPDH- specific products of 205 and 148 bp, respectively.
(TIF)

S2 Fig *mab21l2* compound heterozygous embryos generated by the cross of c.150_156delCCGTTTC, p.(Arg51Serfs*4) and c.151dupC, p.(Arg51Profs*14) heterozygous carriers. A wild-type fish from the same progeny is shown at the top and followed by 7 compound heterozygous embryos. Please note small eye (black arrowheads) in all mutant embryos and shortened tail (black arrows) in 4 out of 7 fish.
(TIF)

S3 Fig TUNEL results in 48–72-hpf wild-type (A-D), *mab21l2* ^Q48Sfs*5^ embryos (E-H) and *mab21l2* ^*R51_F52del*^ mutants (I-L) are shown. An increase in TUNEL staining was observed in both *mab21l2* mutants with remarkably high levels in the *mab21l2* ^Q48Sfs*5^ embryos (E-H) and moderately increased levels in the *mab21l2* ^*R51_F52del*^ embryos (I-L); arrowheads indicate sites of increased TUNEL staining in the eye and brain; le, lens; r, retina; m, midbrain.
(TIF)

S4 Fig Immunostaining with PCNA (Proliferating Cell Nuclear Antigen) and DAPI in 24–72-hpf wild-type (A-F), *mab21l2* ^Q48Sfs*5^ (A1-F1), and *mab21l2* ^*R51_F52del*^ (A2-F2) embryos. Overlay images of the PCNA and DAPI immunostaining are shown in Fig. 6. The arrowheads in A1 and A2 indicate abnormal retinal folding;le, lens; m, midbrain; r, retina.
(TIF)

S5 Fig Immunostaining with ZL-1 (red) and in situ hybridization with *pax6b*, *pax2*.*1* and *foxe3* antisense riboprobes in wild-type (A-F), *mab21l2* ^Q48Sfs*5^ (A1-F1) and *mab21l2* ^*R51_F52del*^ (C2-F2) embryos. The ZL-1 staining is absent in 24-hpf (A1) but present in 48–72-hpf mutant embryos (B1, C1, C2), *pax2*.*1* pattern is abnormal in 72-hpf mutant embryos (D1, E1, D2, E2), arrowhead in D1 shows abnormal areas of *pax2*.*1*-positive cells in the central retina and arrowheads in E1 show broad and intense expression in the region of optic fissure; *foxe3* expression is decreased in 48-hpf mutants (F1, F2); le, lens; of, optic fissure.
(TIF)
