# Supplementary material for: Mutations in MAB21L2 Result in Ocular Coloboma, Microcornea and Cataracts
Source: PLoS Genet. 2015 Feb 26;11(2):e1005002. doi: 10.1371/journal.pgen.1005002 (PMC4342166; doi:10.1371/journal.pgen.1005002)
Supplement: S1 Table — (DOC) [file pgen.1005002.s001.doc]

**Supplemental Table 1. Summary of novel or rare heterozygous variants predicted to be functionally significant.**

| **Gene 1** | **Transcript 1** | **Variant** | **EVS frequency** | **SNP#** | **HGVS Nucleotide** | **Amino acid** | **Damaging Predictions*** | **GERP++** | **PhyloP** | **BioGPS** | **Co-segregation** |
| --- | --- | --- | --- | --- | --- | --- | --- | --- | --- | --- | --- |
| ***Nonsense, splicing or frameshift*** | | | | | | | | | |  |  |
| *DISP2* | NM_033510 | 15:40657155 | 0/13006 | - | c.719+1G>A | NA | NA | 4.7 | 2.44 | ++ | No |
| *FAM200B* | NM_001145191 | 4:15688985 | 1/6582 | - | c.386_387del | p.Lys129Ilefs*8 | NA | NA | NA | NA | No |
| ***Inframe indels*** | | | | | | | | | |  |  |
| *TCEB3B* | NM_016427 | 18:44560827 | 0/13006 | - | c.792_809del | p.Arg264_Ala269del | NA | NA | NA | + | No |
| ***Novel Missense*** | | | | | | | | | |  |  |
| *DHFR* | NM_000791 | 5:79949838 | 0/12154 | - | c.125C>T | p.Ser42Phe | 5/5 | 5.67 | 2.68 | ++ | No |
| ***MAB21L2*** | **NM_006439** | **4:151504332** | **0/13006** | **-** | **c.151C>G** | **p.Arg51Gly** | **4/5** | **6.16** | **2.94** | **++++** | **Yes** |
| *PGAP2* | NM_001145438 | 11:3832586 | 0/12998 | - | c.268G>A | p.Ala90Thr | 4/5 | 4.92 | 1.48 | ++ | No |
| ***Rare Missense*** | | | | | | | | | |  |  |
| *ABCC2* | NM_000392 | 10:101594176 | 7/13006 | rs142715085 | c.3298C>T | p.Arg1100Cys | 5/5 | 4.38 | 1.23 | + | ND |
| *ASPM* | NM_018136 | 1:197073196 | 122/13002 | rs41299623 | c.5185C>T | p.Arg1729Trp | 4/5 | 5.98 | 2.84 | ++ | ND |
| *KCNG4* | NM_172347 | 16:84256128 | 66/13000 | rs140124801 | c.1255G>A | p.Val419Met | 5/5 | 5.61 | 2.63 | + | ND |
| *KIF25* | NM_030615 | 6:168443258 | 1/13006 | - | c.847G>A | p.Gly283Arg | 4/5 | 4.27 | 2.09 | + | ND |
| *NNT* | NM_012343 | 5:43700321 | 56/13006 | rs78818665 | c.2977A>G | p.Ile993Val | 5/5 | 5.76 | 2.2 | + | ND |
| *PIWIL3* | NM_001008496 | 22:25124155 | 45/13006 | rs148034582 | c.1921G>A | p.Val641Met | 4/5 | 1.68 | 0.5 | + | ND |
| *PKN2* | NM_006256 | 1:89299123 | 3/12320 | - | c.2947T>C | p.Trp983Arg | 4/5 | 5.84 | 2.23 | ++ | ND |
| *SI* | NM_001041 | 3:164764786 | 13/13006 | rs121912615 | c.1730T>G | p.Val577Gly | 5/5 | 5.36 | 2.04 | - | ND |
| *SPATA17* | NM_138796 | 1:217955553 | 8/13004 | rs145637899 | c.761G>A | p.Arg254His | 4/5 | 4.73 | 2.35 | + | ND |
| *SLC5A4* | NM_014227 | 22:32616985 | 17/13006 | rs139861447 | c.1690C>T | p.Arg564Trp | 4/5 | 3.34 | 1.05 | + | ND |

* based on the following 5 effect prediction programs: SIFT, Polyphen2, Mutation Taster, MutationAssessor, and FATHMM

NA- not available; ND- not determined
