# Supplementary material for: Mutations in MAB21L2 Result in Ocular Coloboma, Microcornea and Cataracts
Source: PLoS Genet. 2015 Feb 26;11(2):e1005002. doi: 10.1371/journal.pgen.1005002 (PMC4342166; doi:10.1371/journal.pgen.1005002)
Supplement: S2 Table — (DOCX) [file pgen.1005002.s002.docx]

**Supplemental Table 2.** PCR conditions and oligonucleotides utilized for amplification of gene regions in this study.

| **Gene** | **Sequence (5’→3’)** | **Annealing Temp.** | **Product size** |
| --- | --- | --- | --- |
| ***Human primers to amplify the region around c.151 C>G p.(Arg51Gly) allele*** | | | |
| *MAB21L2* | AGACGCCGGTGTATAGCC (forward) | 60°C | 415 bp |
| *MAB21L2* | TTACGCGCTGAGAGATAGCC (reverse) | 60°C |  |
| ***Human primers to amplify full coding region of MAB21L2*** | | | |
| *MAB21L2* | ccaggtggaaaacgagagtg (forward) | 60°C | 1362 bp |
| *MAB21L2* | gaaggtcttctgccggattc (reverse) | 60°C |  |
| *MAB21L2* | CGGTGGACAAGTGCAGCTAT (internal 1) | N/A |  |
| *MAB21L2* | AGGGATGTGGGGCATAGG (internal 2) | N/A |  |
| ***Zebrafish primers for genotyping of TALEN alleles*** | | | |
| *mab21l2* | TCTTTTCCTGGGAGTTGTGC (forward) | 60°C | 350 bp |
| *mab21l2* | CCCCATCTGGTTCAGGTAAA (reverse) | 60°C |  |
| ***Zebrafish primers surrounding the region utilized for in situ probe*** | | | |
| *mab21l2* | GGGACGTGGTTAAGATGGTG (forward) | 60°C | 698 bp |
| *mab21l2* | GCAAACGTCCGTAAATAGGC (reverse) | 60°C |  |
| ***WES Confirmation Primers for other genes*** | | | |
| *DISP2* | CTTCCCTGCTCTGAGGAGTC (forward) | 60°C | 389 bp |
| *DISP2* | TATGCCAGGCACCATTAGG (reverse) | 60°C |  |
| *FAM200B* | AAGTAAGTGCAAGACGTTATAATGAAG (forward) | 60°C | 338 bp |
| *FAM200B* | TTTTTCAGCAGCTGTGTTAGC (reverse) | 60°C |  |
| *TCEB3B* | GGCCTCTGCTGTGTCCAG (forward) | 60°C | 382 bp |
| *TCEB3B* | CCGTGTGTCCCATTTCCTG (reverse) | 60°C |  |
| *DHFR* | AGGCCCAGTTGCTGATTTC (forward) | 60°C | 400 bp |
| *DHFR* | TTTTAATAATGGTAAATGAACCTTGG (reverse) | 60°C |  |
| *PGAP2* | GACTGAGCACAAGGGCTGAC (forward) | 60°C | 376 bp |
| *PGAP2* | CAGCCCCGTATGTTTGATG (reverse) | 60°C |  |
