# Supplementary figures and images for: Mutations in MAB21L2 Result in Ocular Coloboma, Microcornea and Cataracts
Source: PLoS Genet. 2015 Feb 26;11(2):e1005002. doi: 10.1371/journal.pgen.1005002 (PMC4342166; doi:10.1371/journal.pgen.1005002)

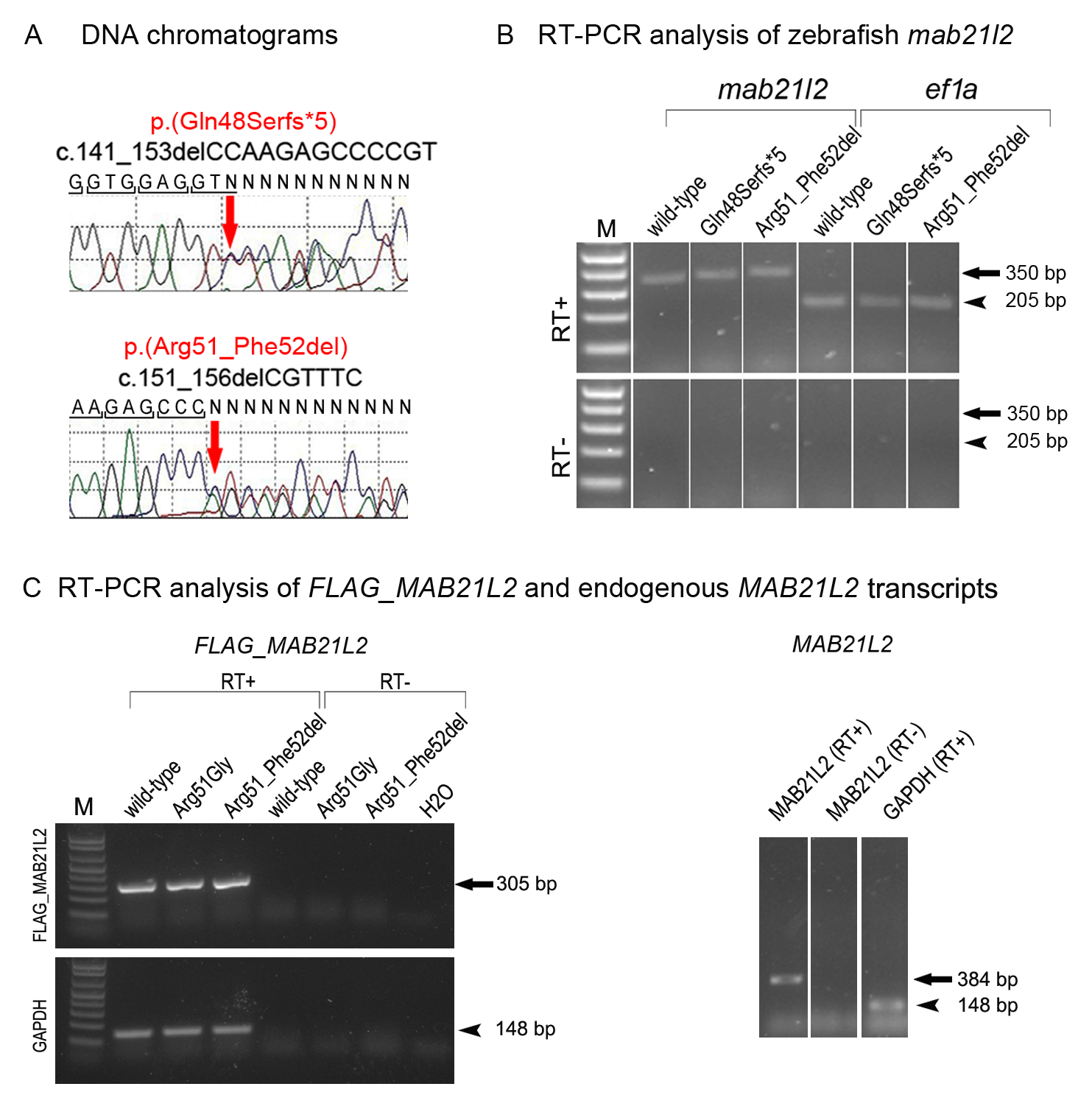

Supplement: S1 Fig — (B) Agarose gel analysis of zebrafish mab21l2 transcript- specific amplification from 48-hpf homozygous zebrafish wild-type and mutant embryos; (C) Agarose gels representing RT-PCR expression analysis of recombinant FLAG-MAB21L2 wild-type and mutant transcripts (left) as well as endogenous MAB21L2 transcript in human lens epithelial cells (HLE-B3). RT minus (RT-) control refers to a mock reverse transcription containing all the RT-PCR reagents, except the reverse transcriptase, to demonstrate the absence of contaminating DNA in the corresponding RNA samples; ef1a and GAPDH amplification was performed for loading control in zebrafish and human samples correspondingly; the reactions were run on the same gel but irrelevant lanes were removed when needed; M- molecular marker, black arrow indicates mab21l2-, FLAG-MAB21L2 or MAB21L2-specific bands of 350, 305 and 384 bp, correspondingly; black arrowhead points to ef1a- and GAPDH- specific products of 205 and 148 bp, respectively. (TIF) [file pgen.1005002.s003.tif]

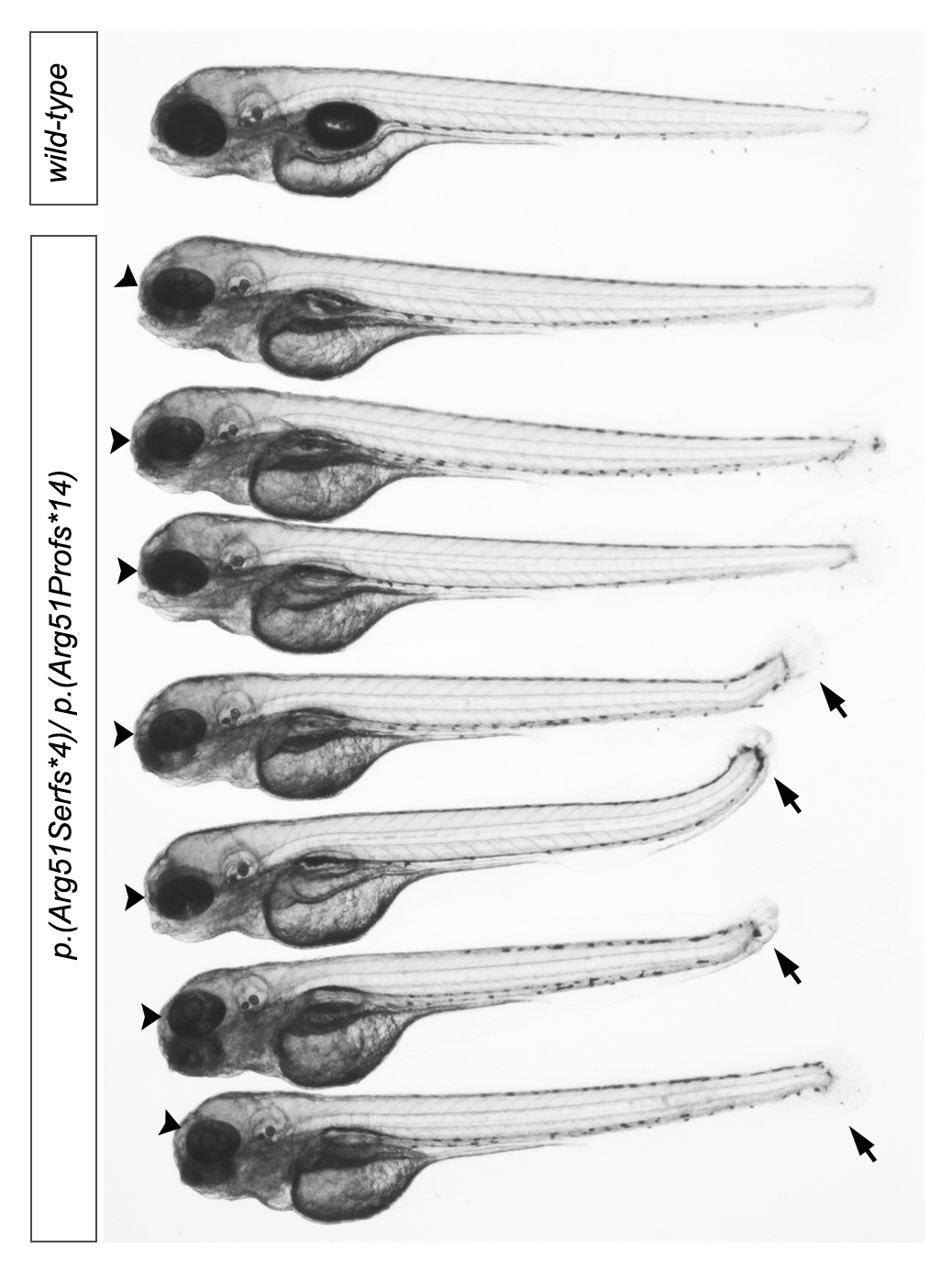

Supplement: S2 Fig — A wild-type fish from the same progeny is shown at the top and followed by 7 compound heterozygous embryos. Please note small eye (black arrowheads) in all mutant embryos and shortened tail (black arrows) in 4 out of 7 fish. (TIF) [file pgen.1005002.s004.tif]

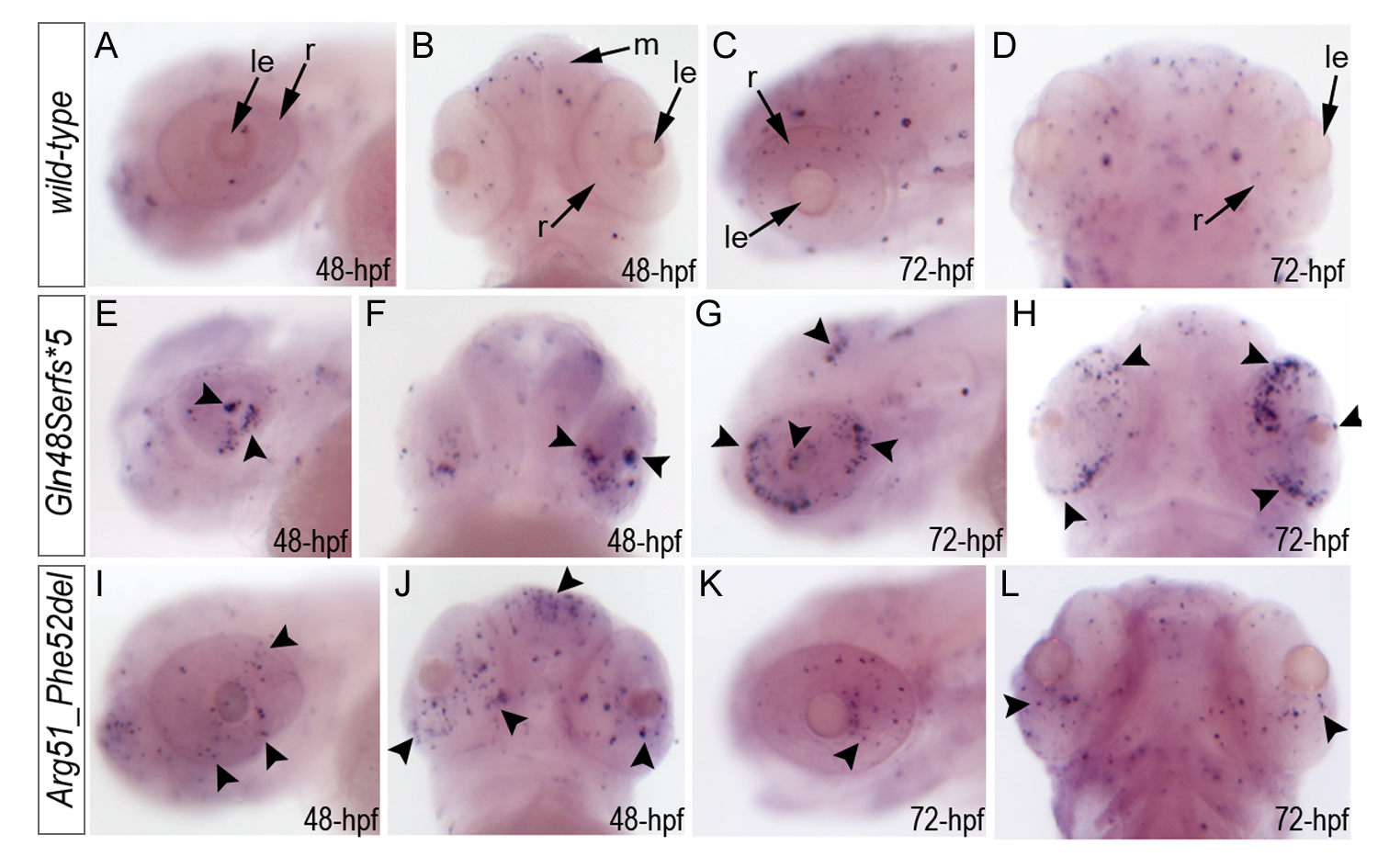

Supplement: S3 Fig — An increase in TUNEL staining was observed in both mab21l2 mutants with remarkably high levels in the mab21l2 Q48Sfs*5 embryos (E-H) and moderately increased levels in the mab21l2 R51_F52del embryos (I-L); arrowheads indicate sites of increased TUNEL staining in the eye and brain; le, lens; r, retina; m, midbrain. (TIF) [file pgen.1005002.s005.tif]

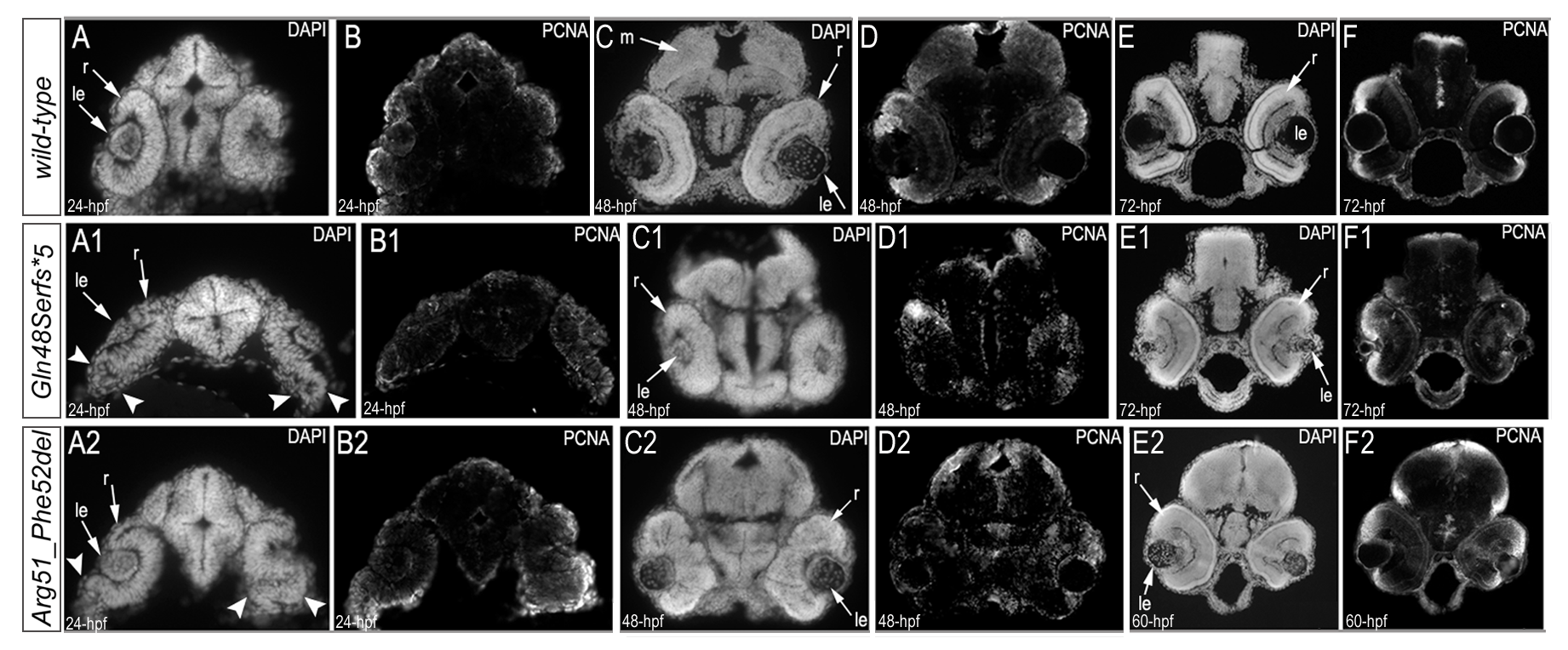

Supplement: S4 Fig — Overlay images of the PCNA and DAPI immunostaining are shown in Fig. 6. The arrowheads in A1 and A2 indicate abnormal retinal folding;le, lens; m, midbrain; r, retina. (TIF) [file pgen.1005002.s006.tif]

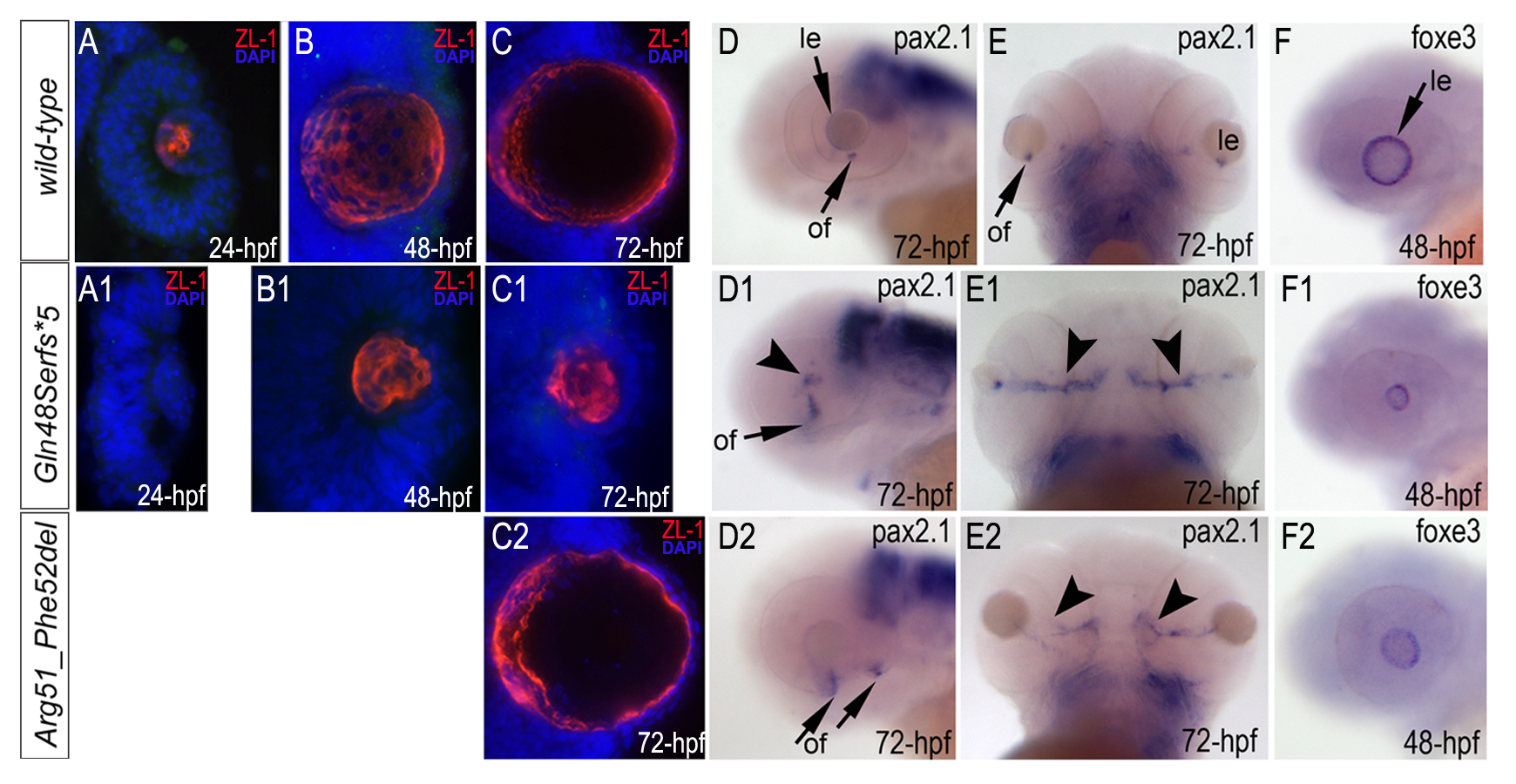

Supplement: S5 Fig — The ZL-1 staining is absent in 24-hpf (A1) but present in 48–72-hpf mutant embryos (B1, C1, C2), pax2.1 pattern is abnormal in 72-hpf mutant embryos (D1, E1, D2, E2), arrowhead in D1 shows abnormal areas of pax2.1-positive cells in the central retina and arrowheads in E1 show broad and intense expression in the region of optic fissure; foxe3 expression is decreased in 48-hpf mutants (F1, F2); le, lens; of, optic fissure. (TIF) [file pgen.1005002.s007.tif]
